# Evaluation of the Dual Impact of Nanotechnologies on Health and Environment Through Alternative Bridging Models

**DOI:** 10.1002/adhm.202505218

**Published:** 2026-01-21

**Authors:** Marie Celine Lefevre, Margherita Bernardeschi, Matteo Battaglini, Gianni Ciofani

**Affiliations:** ^1^ Istituto Italiano di Tecnologia Smart Bio‐Interfaces Pontedera Italy

**Keywords:** alternative models, ecotoxicology, nanomedicine, nanotechnology, translational research

## Abstract

Nanotechnology holds substantial promise across medicine, industry, and environmental science, yet its rapid advancement raises important concerns about potential impacts on both human health and ecosystems. This review explores evidence on alternative bridging models that connect cellular responses to organism‐level outcomes, enabling integrated evaluation of nanomaterial safety and efficacy. Beyond conventional in vitro and mammalian systems, we focus on a diverse range of invertebrates, including *Hydra spp*., planarians, *Caenorhabditis elegans*, *Drosophila melanogaster*, *Bombyx mori*, and bivalve mollusks, and small vertebrates, notably zebrafish and avian embryos. These ethically compliant, cost‐effective, and biologically relevant platforms have been compared regarding the main experimental readouts they provide, from survival and regeneration to behavior, metabolism, and molecular stress responses. Across studies, applications in nanotoxicology and ecotoxicology reveal conserved mechanisms of nanoparticle action, including oxidative stress, genotoxicity, inflammation, and immune modulation, while clarifying links between environmental exposure and human health. In parallel, these models advance nanomedicine development, improving our understanding of biodistribution, targeting precision, and therapeutic efficacy. Finally, we discuss their current limitations, such as a lack of standardization, and propose practical considerations for model selection to strengthen risk assessment, support safer‐by‐design strategies, and promote the responsible development of next‐generation nanotechnologies that balance innovation with sustainability.

## Graphical abstract

This review explores how alternative invertebrate and small‐vertebrate models advance the evaluation of nanomaterials across medicine and environmental science. By bridging cellular and organismal levels, these models enable integrated assessment of toxicity, biodistribution, and therapeutic performance. They provide ethical, cost‐effective, and predictive tools that link environmental safety with human health in the development of sustainable nanotechnologies.

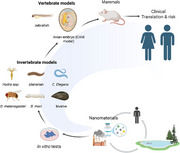

## 1 Introduction: Nanotechnology, a Double‐Edged Sword for Human Health

### 1.1 The Expanding Role of Nanoparticles in Medicine and Industry

Over the decades, nanotechnology has revolutionized the fields of materials science, medicine, energy, and environmental engineering, marking the emergence of functional materials designed at the scale of atoms and molecules [1]. Nanomaterials (NMs), typically defined as materials with at least one dimension between 1 and 100 nanometers, exhibit unique physicochemical properties that differ fundamentally from their bulk counterparts [2, 3]. This category encompasses various nanoscale architectures, including discrete nanoparticles (NPs), as well as nanofibers, nanosheets, and nanocomposites [4]. These novel characteristics, including high surface area‐to‐volume ratios, quantum effects, and enhanced chemical reactivity, have enabled breakthroughs across sectors ranging from targeted drug delivery and biosensing to agriculture, electronics, and environmental remediation [5, 6, 7]. The diversity of engineered nanomaterials (ENMs) reflects their vast technological potential [2, 8, 9]. Metal and metal oxide NPs (e.g., Ag, Au, TiO_2_, ZnO, CeO_2_) are used for their catalytic, antimicrobial, and optical properties [10, 11, 12]. Carbon‐based nanostructures such as graphene, carbon nanotubes, and fullerenes provide exceptional conductivity and mechanical resistance [10]. Polymeric and lipid NPs enable encapsulation and controlled release of bioactive compounds [13], while quantum dots (QDs) and semiconductor nanocrystals offer precise light emission for imaging and sensing [14]. Hybrid nanocomposites, which combine organic and inorganic components, further expand functional possibilities by integrating multiple responses, mechanical, optical, magnetic, or biological, within a single platform [15].

In medicine, NPs have become essential tools for diagnosis and therapy. They act as drug carriers, contrast agents, and multifunctional therapeutic systems capable of crossing biological barriers and releasing drugs at targeted sites [16]. Lipid NPs have revolutionized vaccine delivery [17, 18], while Au and magnetic NPs are used in hyperthermia treatments, biosensing, and targeted imaging [19, 20, 21]. Beyond medicine, they contribute to industrial and agricultural innovation: they enhance coatings, textiles, and cosmetics, improve catalytic efficiency, optimize fertilizers and pesticides, and contribute to renewable energy systems such as fuel cells and solar panels [13, 22, 23]. Collectively, these applications underscore nanotechnology's potential to contribute to sustainability, precision healthcare, and technological innovation. However, the same nanoscale features that make these materials so valuable also raise concerns about their potential toxicological and ecological impacts.

### 1.2 Potential Risks for Human Health

The enhanced surface activity and small size that confer superior functionality may also increase the likelihood of interactions with biological molecules, cells, and tissues [24, 25, 26]. NPs can enter the body through multiple exposure routes, including inhalation, ingestion, dermal penetration, or injection, and their minute dimensions enable translocation across physiological barriers such as the alveolar epithelium, intestinal mucosa, placental barrier, and even the blood–brain barrier [16, 27, 28]. This capacity for systemic distribution increases the likelihood of cellular and molecular interactions. Once internalized, they may accumulate in organs, interfere with subcellular components, and induce biochemical disturbances, including oxidative stress, DNA damage, protein misfolding, inflammation, and apoptosis [29, 30, 31, 32].

Metallic NPs, such as Ag, CuO, or TiO_2_, are known to generate reactive oxygen species (ROS), leading to lipid peroxidation, DNA damage, and protein misfolding [33, 34, 35], whereas carbon nanotubes may induce fibrotic or granulomatous responses resembling asbestos‐like pathology [36, 37, 38]. NPs used in drug carriers may also undergo surface modification or degradation inside the body, leading to altered pharmacokinetics and bioaccumulation [39, 40]. At the molecular scale, NP exposure has been linked to epigenetic alterations, including DNA methylation, histone modification, and microRNA dysregulation, potentially influencing gene expression long after exposure [41, 42, 43, 44]. Their ability to cross the placental barrier and the blood–brain barrier raises additional concerns regarding developmental and neurological toxicity [45]. Such effects have been reported in numerous in vitro and in vivo studies, yet translating these findings into realistic human or environmental risk assessments remains challenging due to the extraordinary diversity of NMs and the complexity of their transformations in biological and ecological systems [46, 47, 48, 49]. Beyond direct effects on human health, the environmental footprint of nanotechnology has become a growing focus of research [50, 51].

### 1.3 Environmental and Ecological Considerations

The rapid expansion of nanotechnology has led to the inevitable release of NPs into air, soil, and aquatic environments, either unintentionally through industrial waste streams or deliberately through applications in agriculture and water treatment. Once in the environment, NPs can interact with biotic and abiotic components, altering their fate, transport, and toxicity [52, 53]. For example, Ag NPs, widely used for their antimicrobial activity, may dissolve to release toxic ionic Ag that disrupts microbial communities and aquatic food webs [54, 55]. Similarly, TiO_2_ and ZnO NPs, common in sunscreens and coatings, can accumulate in sediments and generate photoreactive oxidative species under sunlight, impairing phytoplankton and invertebrates [12]. Once incorporated into trophic chains, NMs and their transformation products may bioaccumulate and biomagnify, indirectly affecting higher organisms [12]. Such environmental perturbations ultimately converge with human exposure routes, through food, water, or occupational contact, highlighting the interconnected nature of ecological and human health. Yet many of these studies rely on vertebrate models, raising ethical and regulatory concerns about animal use, especially given the high number of materials requiring evaluation. Consequently, there is an urgent need for alternative testing strategies that combine biological relevance, translational value, and ethical acceptability to support both human health and environmental risk assessment [56]. Addressing these multifaceted effects requires reliable biological systems capable of linking mechanistic understanding to organism‐level outcomes. Traditionally, such assessments have relied on a combination of in vitro and in vivo models, each offering distinct but limited perspectives.

### 1.4 Conventional Biological Systems for the Study of Benefits and Risks of Nanoparticles

In vitro models based on 2D monolayer cultures are widely used for high‐throughput screening of cytotoxicity, oxidative stress, genotoxicity, and inflammatory responses [57]. These systems allow precise control of experimental variables such as dose, exposure time, and medium composition, and provide biological insight into NP–cell interactions while minimizing ethical concerns. However, conventional 2D assays poorly recapitulate tissue organization, fluid dynamics, and extracellular matrix complexity, resulting in limited predictive value for organism‐level outcomes [58].

To address these limitations, more sophisticated 3D models have emerged, including multicellular spheroids, organoids, and microfluidic “organ‐on‐chip” platforms [59]. These systems recreate structural and biochemical gradients, enabling more realistic exposure scenarios and drug penetration profiles. 3D liver, lung, and intestinal models, for instance, can mimic NP uptake, biotransformation, and barrier crossing under physiologically relevant flow conditions [60, 61, 62, 63]. Yet despite their improved physiological fidelity, these models still fall short in capturing whole‐organism phenomena such as metabolism, clearance, immune activation, and long‐term bioaccumulation—processes that critically determine NP safety and efficacy [11].

In vivo models, most commonly rodents or other vertebrates, remain the gold standard for assessing integrated biological effects, encompassing pharmacokinetics, biodistribution, immunogenicity, and systemic toxicity [64]. Their use has been instrumental in translating NP‐based therapies from bench to clinic. However, these models are inadequate to meet the scale and diversity of NM testing required today. Conventional studies are time‐consuming, resource‐intensive, and limited by ethical constraints regarding animal welfare [64]. Furthermore, the unique physicochemical behaviors of NPs, such as agglomeration, surface charge variation, and ion dissolution, often complicate the extrapolation of rodent data to environmental species or human exposure scenarios [25, 46]. In this context, alternative animal models constitute an essential component of modern nanotoxicology and nanomedicine, occupying an intermediate space between in vitro cell systems and vertebrate organisms, and providing a whole‐organism context while minimizing ethical concerns.

### 1.5 Aim of the Review

This review provides a critical, integrative assessment of how alternative bridging models, including invertebrates and small vertebrates, advance the evaluation of NM dual impact on human health and the environment. Building on the Sections that follow, we characterize the biological and experimental features of these systems and show how they enable multiscale readouts like survival, development, behavior, regeneration, tissue and hematological changes, and cellular–molecular responses, while linking these endpoints to core mechanisms of NP action, including biodistribution, barrier crossing, oxidative stress, genotoxicity, and immune modulation. We examine their predictive value for mammalian outcomes and their ecological relevance, emphasizing when and why each model is most informative, and we situate their use within ethical and regulatory expectations. Finally, we highlight methodological constraints that limit comparability across studies, like dose metrics, particle characterization and dispersion, exposure routes, and time‐integrated concentrations, and outline practical directions for standardization and model selection across ecotoxicology and nanotherapeutic development. The overarching goal is to provide a framework that aligns experimental findings with translational applications, supporting safer‐by‐design nanotechnologies and more reliable and translatable risk evaluation.

## 2 Alternative Bridging Models

From a biomedical and toxicological perspective, the adoption of alternative animal models represents a major step forward in evaluating NP safety, biodistribution, and therapeutic potential. Rather than serving as replacements for mammalian systems, these models form a complementary experimental continuum that bridges cellular assays and complex vertebrate biology. Collectively, they provide a continuous framework in which simple invertebrates enable rapid biological insights, while small vertebrates recapitulate systemic physiological responses under vertebrate‐relevant conditions.

**FIGURE 1:**
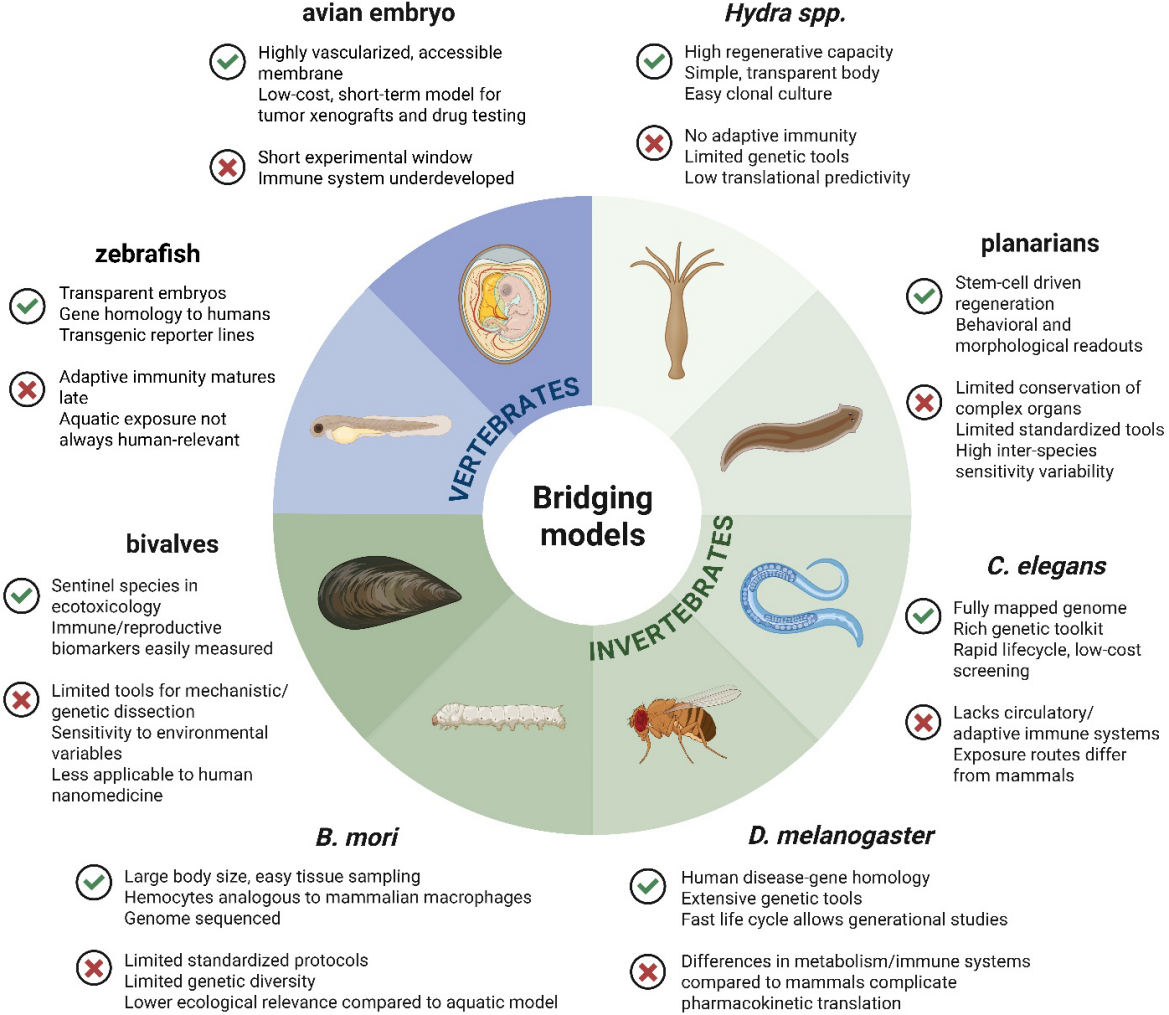
Representative invertebrate models for nanotoxicology and nanomedicine testing, used as alternative systems to assess nanomaterial safety and efficacy, highlighting their biological diversity and translational relevance. Created with BioRender.

Alternative bridging models include a wide range of invertebrate and small‐vertebrate species, including the nematode *Caenorhabditis elegans*, the freshwater cnidarian *Hydra spp*., the fruit fly *Drosophila melanogaster*, the planarian flatworms, the silkworm *Bombyx mori*, marine and freshwater bivalves, avian embryos, and the zebrafish (*Danio rerio*) [65, 66, 67, 68, 69, 70, 71, 72, 73]. Each model contributes a specific niche within this continuum (Figure 1). Simple organisms such as *Hydra spp*. and planarians offer exceptional access to regeneration, morphogenesis, and oxidative stress responses, providing early indicators of cellular disruption by NMs [66, 68]. Intermediate invertebrates such as *C. elegans* and *D. melanogaster* add layers of genetic, behavioral, and neurophysiological complexity, allowing conserved stress, apoptotic, and metabolic pathways to be studied in vivo [65, 67, 74]. *B. mori*, a large invertebrate with accessible hemolymph and immune tissues, extends this framework toward tissue‐level analyses relevant to innate immunity and systemic inflammation [71]. Progressing to vertebrate models such as zebrafish, experimental accessibility is combined with vertebrate‐like organ systems, toxicokinetics, and genetic conservation, permitting the exploration of biodistribution, metabolism, and organ‐specific toxicity under realistic biological conditions [69, 75]. Finally, avian embryos and chorioallantoic membrane (CAM) assays provide a unique preclinical window into vascular interactions, biodistribution, and therapeutic performance, linking nanotoxicology with translational nanomedicine [73].

Framing these species within a biological and functional hierarchy clarifies their complementary strengths and explains their subsequent deployment throughout this review. In the following sections, functional, ecotoxicological, and biomedical case studies are organized according to this progression—from regenerative and cellular models to vertebrate systems capable of capturing toxicokinetics and organ‐level pathophysiology. This structure emphasizes how alternative models collectively inform both environmental safety and human health risk assessment of ENMs.

Beyond their biological complementarity, these models offer major ethical and practical advantages that have driven their adoption in modern nanotoxicology. Their small size, rapid life cycles, and potential for automation make them compatible with large‐scale and comparative NP testing, while their experimental accessibility allows simultaneous evaluation of safety, biodistribution, and functionalization. Importantly, most of these species are not classified as protected “animals used for scientific purposes” under major regulatory frameworks such as the EU Directive 2010/63/EU, OECD guidelines, or the U.S. Toxic Substances Control Act (TSCA), enabling compliance with the 3Rs principles (Replacement, Reduction, and Refinement) [76, 77, 78]. As a result, they provide ethically responsible and cost‐efficient platforms for early hazard identification and preclinical screening, ensuring that efficacy and safety assessments progress in parallel before mammalian validation.

### 2.1 Invertebrate Models

Invertebrate models occupy a central place within the spectrum of alternative systems used in nanotoxicology and nanomedicine. They combine experimental simplicity with remarkable biological diversity, allowing the study of conserved cellular and physiological processes across evolution. Their short life cycles and ease of culture make them particularly suited for detailed biological and multigenerational analyses under controlled conditions. These organisms encompass a broad phylogenetic range, from simple diploblastic species such as *Hydra spp*. to more complex bilaterians, including planarians, nematodes, insects, and bivalves. Collectively, they provide biological insight into fundamental processes such as regeneration, oxidative stress, neurotoxicity, and metabolism, offering an intermediate level of biological organization between cell culture systems and vertebrate models [79, 80]. Some, like marine and freshwater bivalves, also serve as environmentally relevant sentinels, linking laboratory findings to ecosystem‐level implications [81].

*Hydra spp*. are small freshwater cnidarians with a simple tubular body consisting of two epithelial layers surrounding a central gastrovascular cavity [82]. Despite this simplicity, *Hydra spp*. displays continuous self‐renewal from three stem‐cell lineages, granting them an extraordinary ability to fully regenerate lost body parts from small tissue fragments [82]. Their asexual reproduction by budding provides genetically identical clones, facilitating reproducibility and large‐scale culturing under controlled laboratory conditions. Their transparency and defined anatomy allow real‐time imaging of morphological and cellular changes after NP exposure [83]. Alterations in regeneration, epithelial integrity, or oxidative balance serve as sensitive integrative markers of biocompatibility, positioning *Hydra spp*. as a pivotal bridge between simple cellular assays and more complex metazoan models [66]. Planarians are free‐living flatworms widely distributed in freshwater environments, characterized by bilateral symmetry and a dorsoventrally flattened body with a simple cephalic region bearing two eyespots connected to paired ventral nerve cords [84]. Their body architecture supports an extensive population of pluripotent stem cells (neoblasts), which drive full‐body regeneration after injury [84, 85]. This remarkable regenerative capacity makes planarians powerful indicators of NP interference with cell proliferation, differentiation, and morphogenesis. Their sensitivity to environmental stressors, combined with measurable behavioral and oxidative‐stress endpoints, provides a sensitive readout of systemic and neurotoxic effects [68]. Compared with *Hydra spp*., planarians capture stem cell–mediated regeneration within a bilaterian context, offering a relevant model for developmental and genotoxic evaluation of NPs [68, 84].

*Caenorhabditis elegans* is a small free‐living nematode approximately one millimeter long, with a transparent, segmented body containing a simple digestive tract and a well‐mapped nervous system of 302 neurons [86]. Adult hermaphrodites consist of exactly 959 somatic cells organized into specialized tissues, including the epidermis, musculature, intestine, and gonads, allowing detailed anatomical and developmental characterization [87]. The species develops rapidly through four larval stages before reaching adulthood within about three days at 20°C, and its transparency at all life stages enables direct visualization of organs and gene expression in vivo using fluorescent reporters [88]. Its genetic tractability, with extensive mutant and RNAi libraries, facilitates mechanistic dissection of NP effects on survival, reproduction, and locomotion [65, 88]. Because of its conserved stress‐response pathways and defined physiology, *C. elegans* serves as a cost‐effective and ethically compliant in vivo bridge between simple invertebrate assays and vertebrate nanotoxicology [65].

The fruit fly *Drosophila melanogaster* is a holometabolous insect with a segmented body plan composed of a head, thorax, and abdomen, and well‐developed digestive and nervous systems [89]. Its short life cycle of about ten days and external development make it a highly tractable model for genetic and physiological studies [90]. The adult fly possesses a central nervous system with approximately 200 000 neurons, a complex gut, and an open circulatory system that facilitates systemic analyses of toxicity [91, 92]. Its extensive genetic toolkit, including transgenic drivers, mutant libraries, and CRISPR‐based editing, enables tissue‐specific assessment of NP effects on development, metabolism, and neurobiology [74, 92]. Endpoints such as lifespan, reproduction, oxidative stress, and locomotor behavior reveal conserved mechanisms of nanotoxicity relevant to higher organisms [67]. With roughly 75% of human disease‐related genes conserved, *D. melanogaster* serves as a genetically versatile and physiologically relevant intermediary between simple invertebrate and vertebrate models in nanotoxicology [67, 74].

The silkworm *B. mori* is a lepidopteran insect domesticated for silk production with a soft, cylindrical body divided into three thoracic and ten abdominal segments [93]. Its complete metamorphosis, from egg to larva, pupa, and adult moth, offers access to distinct developmental stages with diverse physiological profiles [94]. The hemolymph, midgut, and fat body provide accessible tissues for evaluating NP distribution, immune activation, and metabolic responses under controlled exposure conditions [95]. As an invertebrate model with well‐characterized genetics and predictable developmental timing, *B. mori* allows standardized toxicological testing while remaining ethically compliant [71, 95]. Observed endpoints include growth inhibition, oxidative stress, and immune modulation, reflecting mechanisms relevant to higher organisms [96]. Its size, ease of handling, and similarity of immune responses to vertebrates make *B. mori* an intermediate system bridging in vitro assays and small vertebrate models in nanotoxicology [94, 96].

Bivalves are aquatic mollusks, such as *Mytilus galloprovincialis*, *Mytilus edulis*, and *Ruditapes philippinarum*, characterized by a laterally compressed body enclosed within two calcareous shells joined by a hinge ligament [97]. They are filter‐feeding organisms that continuously process large volumes of water, leading to efficient accumulation of NPs and other contaminants [98]. Their open circulatory system, hemocytes, and gill epithelia provide accessible endpoints for assessing oxidative stress, genotoxicity, immune responses, and biomineralization disturbances [99]. Due to their ecological importance and sensitivity to environmental changes, bivalves are widely used as sentinel species for aquatic nanotoxicology [100]. Their well‐established biomarkers and ability to bioaccumulate NPs make them key models for linking environmental exposure to cellular and molecular mechanisms of NP‐induced stress [100, 101].

### 2.2 “Unconventional” Vertebrates

Taken together, invertebrate models establish the biological foundation of NP toxicology, enabling precise molecular and cellular interrogation. Building on these insights, small vertebrate systems extend the analysis to organ‐level physiology and toxicokinetic processes under vertebrate‐relevant conditions, by providing higher physiological and anatomical similarity to humans while maintaining accessibility and ethical feasibility. They offer complex organ systems, closed circulatory networks, and vertebrate‐specific features, such as adaptive immunity and endocrine regulation, that enable realistic assessment of NP biodistribution, metabolism, and organ‐level toxicity.

Among these, the zebrafish and avian embryos have emerged as the most widely adopted alternatives in nanotoxicology and nanomedicine. Their external development, optical accessibility, and extensive genetic resources allow real‐time visualization of NP uptake, biotransformation, and systemic effects under vertebrate‐relevant conditions. Both models connect early experimental observations with preclinical validation, supporting investigations into developmental, cardiovascular, hepatic, and neurobehavioral toxicity, as well as nanotherapeutic efficacy and targeting [69, 102]. Importantly, early life stages of zebrafish and embryonic avian systems fall outside major vertebrate protection frameworks, enabling ethically compliant experimentation in accordance with the 3Rs principles [102]. Their use facilitates rapid, cost‐effective, and mechanistically informative assessments that complement mammalian studies while reducing the need for higher‐order vertebrates.

The zebrafish is a small tropical freshwater fish widely used in developmental biology and toxicology due to its genetic similarity to mammals and optical transparency during early life stages [103]. The embryo develops externally and shares approximately 70% of human genes, including key pathways involved in oxidative stress, metabolism, and inflammation [104]. Its small size, high fecundity, and rapid development enable high‐throughput screening of NPs under standardized conditions [69]. The transparent chorion and early organogenesis allow real‐time visualization of biodistribution and toxic effects using fluorescent or labeled NMs [69, 103]. Common endpoints include mortality, hatching delay, cardiac and behavioral alterations, and molecular biomarkers of oxidative or genotoxic stress [75]. Because of its vertebrate physiology, conserved organ systems, and suitability for imaging, the zebrafish serves as an essential bridge between invertebrate assays and mammalian models in nanotoxicology and nanomedicine [69, 75, 104, 105].

Avian embryos, particularly chicken (*Gallus gallus*) and quail (*Coturnix japonica*), develop within the egg and form a richly vascularized extra‐embryonic tissue known as the CAM [73, 102]. The CAM arises from the fusion of the chorion and allantois and functions as a respiratory and excretory organ, mediating gas exchange and calcium absorption. Its dense capillary network and easy experimental accessibility make it an excellent platform for studying angiogenesis, inflammation, and NP biodistribution [106]. Because the avian immune system is not fully developed until late embryogenesis, the CAM allows xenografting and implantation of foreign cells, tissues, and materials without immune rejection, enabling direct assessment of host–material interactions [107, 108]. NPs can be applied topically, intravenously, or via the air sac, allowing assessment of biocompatibility, vascular response, and tissue accumulation without the ethical constraints of mammalian models [109]. This model supports real‐time imaging and histological analyses, providing a rapid, low‐cost, and ethically acceptable vertebrate system for preclinical screening. By combining in vivo physiological relevance with experimental simplicity, the CAM assay bridges traditional toxicology with biomedical nanotechnology, complementing zebrafish studies in the vertebrate spectrum [106].

### 2.3 Comparative Overview of Alternative Models in Nanotoxicology and Nanomedicine

Together, the introduced alternative models illustrate how evolutionary diversity can be harnessed to build a complementary and ethically sustainable experimental framework for NM testing. Simpler invertebrates such as *Hydra spp*. and planarians enable rapid, mechanistic analyses of cellular and regenerative toxicity, while intermediate species like *C. elegans*, *D. melanogaster*, and *B. mori* introduce genetic and physiological complexity relevant to multicellular organisms. Bivalves extend this perspective to environmentally relevant exposure scenarios, whereas zebrafish and avian embryos capture vertebrate‐level toxicokinetics, organ specificity, and translational nanomedical responses. Each model, therefore, contributes distinct insights according to its biological organization and experimental tractability, and their combined use provides a multiscale understanding of NP behavior from environmental safety to biomedical efficacy. While Figure 1 provides a conceptual overview of the biological continuum linking invertebrate and vertebrate models, Table 1 summarizes their main experimental strengths, limitations, and representative applications in nanotoxicology and nanomedicine.

**TABLE 1:** Comparative overview of alternative models used in nanotoxicology and nanomedicine.

Model	Main strengths	Limitations	Applications
*Hydra* spp.	Simple anatomy; continuous stem‐cell renewal; high regenerative capacity; transparency for imaging	Lacks complex organs and adaptive responses	Early screening of cytotoxicity; oxidative stress; epithelial integrity
Planarians	Pluripotent neoblasts; regeneration of complex tissues; behavioral endpoints	Limited genetic tools; aquatic culture constraints	Developmental and regenerative toxicity; neurotoxicity assessment
*C. elegans*	Fully mapped genome and nervous system; transparency; high‐throughput assays	No circulatory system; limited metabolic diversity	Mechanistic toxicology; oxidative stress; neurobehavior; multigenerational effects
*D. melanogaster*	Extensive genetic toolkit; conserved disease pathways; systemic endpoints	Short lifespan; open circulation; limited exposure routes	Developmental, metabolic, and neurobehavioral nanotoxicology; genetic screening
*B. mori*	Large size; accessible tissues (hemolymph, midgut); immune parallels with vertebrates	Labor‐intensive rearing; incomplete genetic resources	Immune and metabolic effects; biodistribution; systemic inflammation
Bivalves	Ecological relevance; filter‐feeding bioaccumulation; established biomarkers	Limited genetic manipulation; variable field conditions	Ecotoxicology; environmental monitoring; bioaccumulation studies
Zebrafish	Vertebrate anatomy; transparency of embryos; genetic homology (∼70% human genes)	Limited adaptive immunity in early stages; aquatic exposure only	Organ‐level toxicity, biodistribution, pharmacokinetics, and nanotherapeutic efficacy
Avian embryo / CAM assay	Highly vascularized tissue; permits xenografting; direct NP delivery; imaging access	Short experimental window; limited systemic endpoints	Angiogenesis; tumor targeting; biodistribution; biocompatibility testing

Collectively, these models form an integrative experimental continuum that captures NP interactions from the cellular to the organismal scale. Building on this framework, the next section explores how these systems are applied to evaluate NP impacts in environmental contexts, focusing on their role as sentinels for ecotoxicological risk assessment.

## 3 Alternative Bridging Model for Ecotoxicology Studies

**FIGURE 2:**
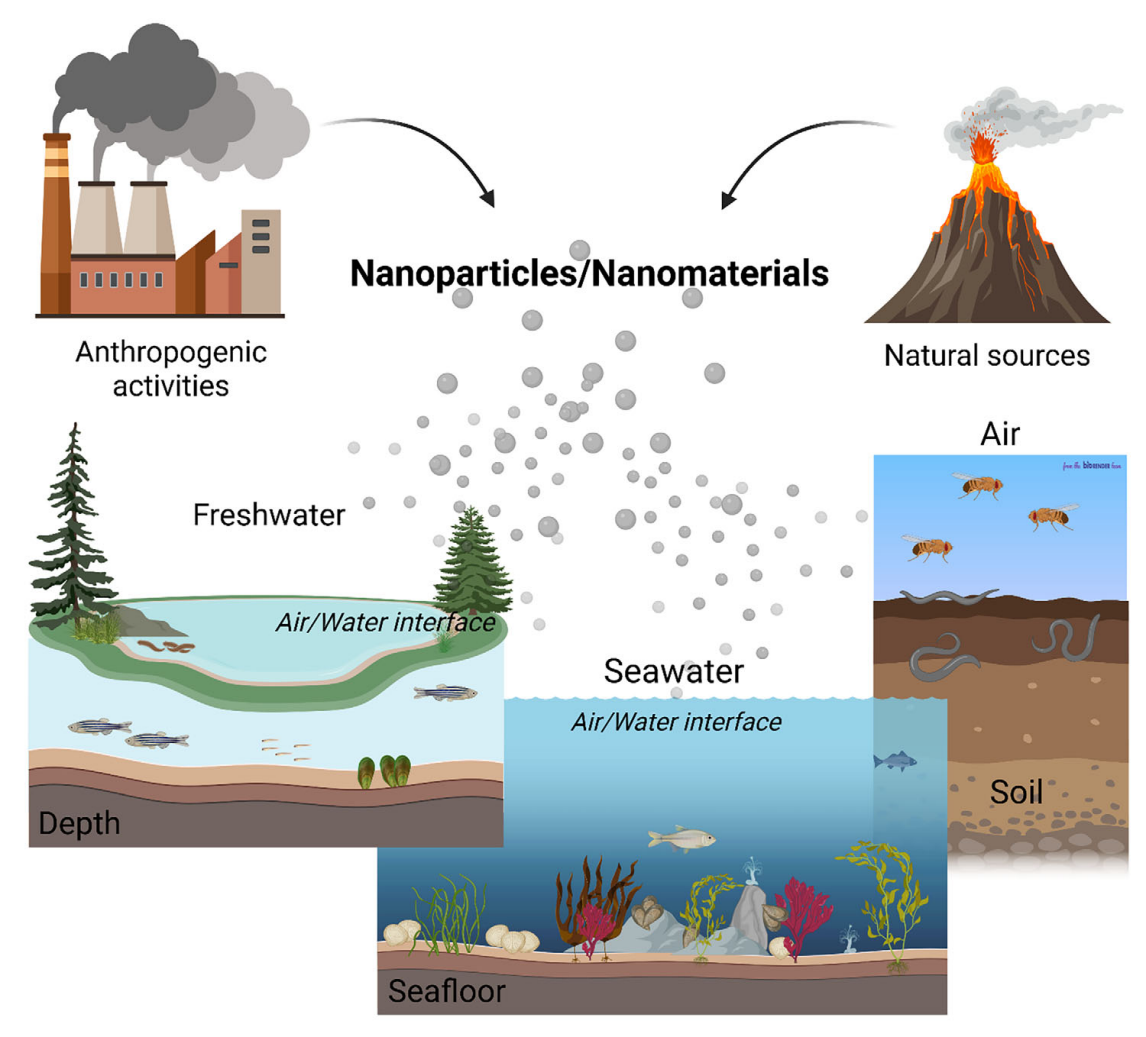
Overview of aquatic and terrestrial organisms employed to investigate environmental NP impacts, showing connections among freshwater, marine, and sediment compartments and how these models bridge ecological and human health risk assessment. Created with BioRender.

**FIGURE 3:**
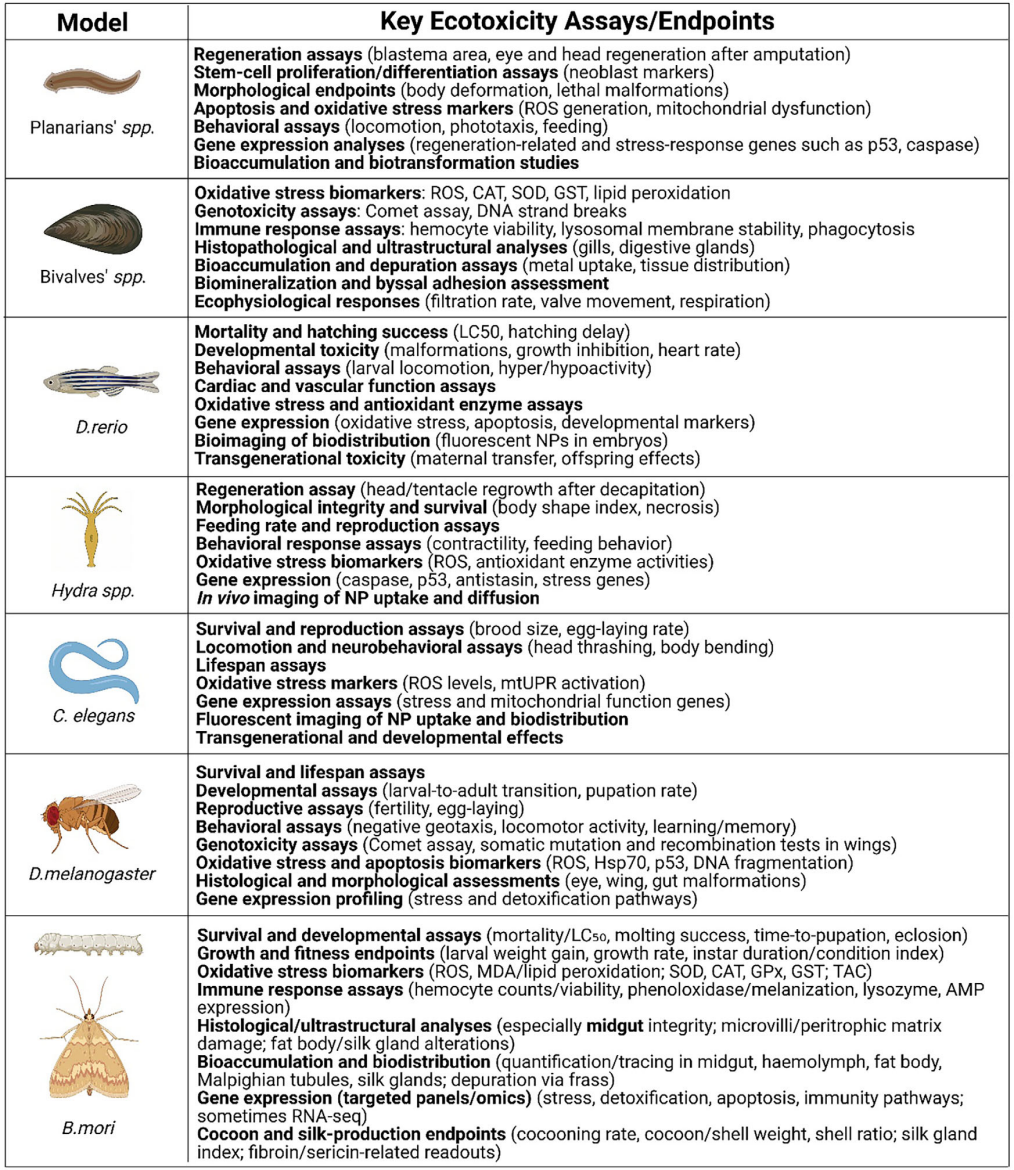
Overview of the bridging model species considered in the ecotoxicological studies included in this review. For each model, the key ecotoxicity assays and analyzed endpoints are reported. Created with BioRender.

While the exchange of matter and energy among environmental compartments, through processes such as water flow, particle transport, and chemical reactions, is well understood, the equally important biological connections among the organisms inhabiting these systems, from population interactions to molecular communication, are often overlooked. (Figure 2). As extensively discussed in the previous sections, even though humans are phylogenetically very distant from most organisms colonizing diverse ecosystems, certain features have remained highly conserved. For this reason, it is particularly interesting to investigate how these anatomical and physiological similarities can serve as a “bridge” in biomedical research. After an initial overview of the main natural environments considered (freshwater, marine, and soil), we will explore the use of various model organisms in ecotoxicological studies concerning exposure to NPs/NMs of different types, always keeping in focus their relevance for assessing potential effects on human health (Figure 3).

### 3.1 Toxicological Effects of NM Exposure

A growing body of ecotoxicological research indicates that ENMs, including metal/metal‐oxide NPs (e.g., Ag, ZnO, TiO_2_), carbon‐based NMs, and nanoplastics, can elicit significant biological responses in freshwater organisms. Across numerous studies, ENMs are shown to be rapidly taken up and capable of disrupting physiological and biochemical homeostasis, often through oxidative stress–linked mechanisms. Common endpoints include ROS generation, alterations in antioxidant enzyme activity (e.g., superoxide‐dismutase ‐SOD‐, catalase ‐CAT‐, glutathione S‐transferase ‐GST‐), lipid peroxidation, DNA damage (assessed through Comet assay, TUNEL), impaired feeding or filtration, reduced growth, and developmental delays or mortality [110, 111, 112]. Mechanistically, freshwater studies often highlight two overlapping modes of action: (i) particle‐specific effects, such as mechanical damage and cellular uptake, and (ii) ion‐mediated toxicity due to partial dissolution of metallic components (e.g., Ag^+^, Zn^2^^+^). Multiple comparative studies employing nanoparticulate vs. ionic or bulk material controls help delineate these toxicity pathways [113, 114]. Variability across freshwater studies is substantial, reflecting differences in particle chemistry, size, surface coatings, aggregation behavior, and exposure scenarios (acute vs. chronic; waterborne vs. dietary). While some investigations report acute toxicity or developmental arrest at high concentrations, others find only sublethal biomarker changes at environmentally realistic levels [115]. Additionally, environmental modifiers—such as ionic strength, natural organic matter, and temperature—alter particle fate (e.g., aggregation, dissolution) and thus toxicity outcomes, complicating direct comparison across freshwater systems [110, 114].

Marine and coastal studies reveal many of the same biological outcomes observed in freshwater systems, including oxidative stress, bioaccumulation, impaired feeding or adhesion (e.g., byssal thread production in bivalves), and reproductive and developmental toxicity. However, marine studies introduce additional complexity due to the physicochemical characteristics of seawater. The high ionic strength and prevalence of bivalent cations (e.g., Ca^2^^+^, Mg^2^^+^) in marine systems promote rapid particle aggregation and surface charge modifications, which may reduce particle dispersion but enhance ingestion by filter feeders [116, 117]. Marine ecotoxicology also places greater emphasis on trophic transfer, bioaccumulation in edible tissues, and the implications for seafood safety—areas less commonly addressed in freshwater studies [116, 118]. In marine bivalves and other taxa, nanoplastics (e.g., polystyrene ‐PS‐) and metal‐based ENMs accumulate in gills, digestive glands, and gonads, inducing tissue‐specific oxidative and genotoxic responses. These effects are strongly mediated by particle aggregation and dispersion protocols, which vary widely among studies and influence uptake [119, 120]. Species‐specific sensitivity is another source of variability. Suspension‐feeding bivalves (e.g., *Mytilus spp*.) exhibit clear sublethal responses—such as lipid peroxidation and altered enzyme activity—while early life stages or mobile species show different profiles, including behavioral disruptions and altered hatching [116]. Several studies further incorporate environmental stressors (e.g., temperature, hypoxia), demonstrating interactive effects that often amplify NM toxicity [116, 118].

A subset of the literature focuses on benthic compartments, sedimentation, and food web dynamics. Due to their aggregation or higher density, many ENMs and nanoplastics preferentially settle into sediments, where they become available to deposit‐feeding and benthic organisms. These taxa can act as vectors for upward trophic transfer, with studies documenting particle retention in gut contents, feces/pseudofeces, and variable tissue bioaccumulation [114, 116]. While evidence for biomagnification remains limited, ecological redistribution is well supported. Differences across sediment studies arise from variations in particle type (e.g., polymer vs. metal oxide), sediment‐mediated transformations (e.g., sulfidation, eco‐corona formation), and exposure duration. Some studies report adverse effects in predators due to trophic transfer, though outcomes are inconsistent and depend on study design. Across all environments, there is increasing adoption of robust physicochemical characterization tools (e.g., dynamic light scattering, zeta potential, transmission electron microscopy ‐TEM‐), use of oxidative and genotoxic biomarkers, and—where possible—direct particle tracking in tissues via techniques like single‐particle inductively‐coupled plasma mass spectrometry (spICP‐MS), TEM, or fluorescent labeling [113, 119]. However, limitations persist due to non‐standardized dispersion protocols, inconsistent exposure concentration measurements (e.g., lack of time‐integrated metrics like AUC), and uneven use of appropriate controls (ionic or bulk forms). These issues hinder data comparability across studies and species.

### 3.2 Bridging Environmental and Human Health

The ecotoxicological literature on NMs consistently demonstrates that toxicity is highly context‐dependent, shaped by the interaction between particle‐specific properties (e.g., chemistry, size, surface coating) and environmental variables (e.g., salinity, temperature, natural organic matter, oxygen availability) [121, 122, 123]. This complexity poses significant challenges for extrapolating findings across ecosystems—and particularly for linking environmental effects to potential human health risks.

#### 3.2.1 Hydra spp

**FIGURE 4:**
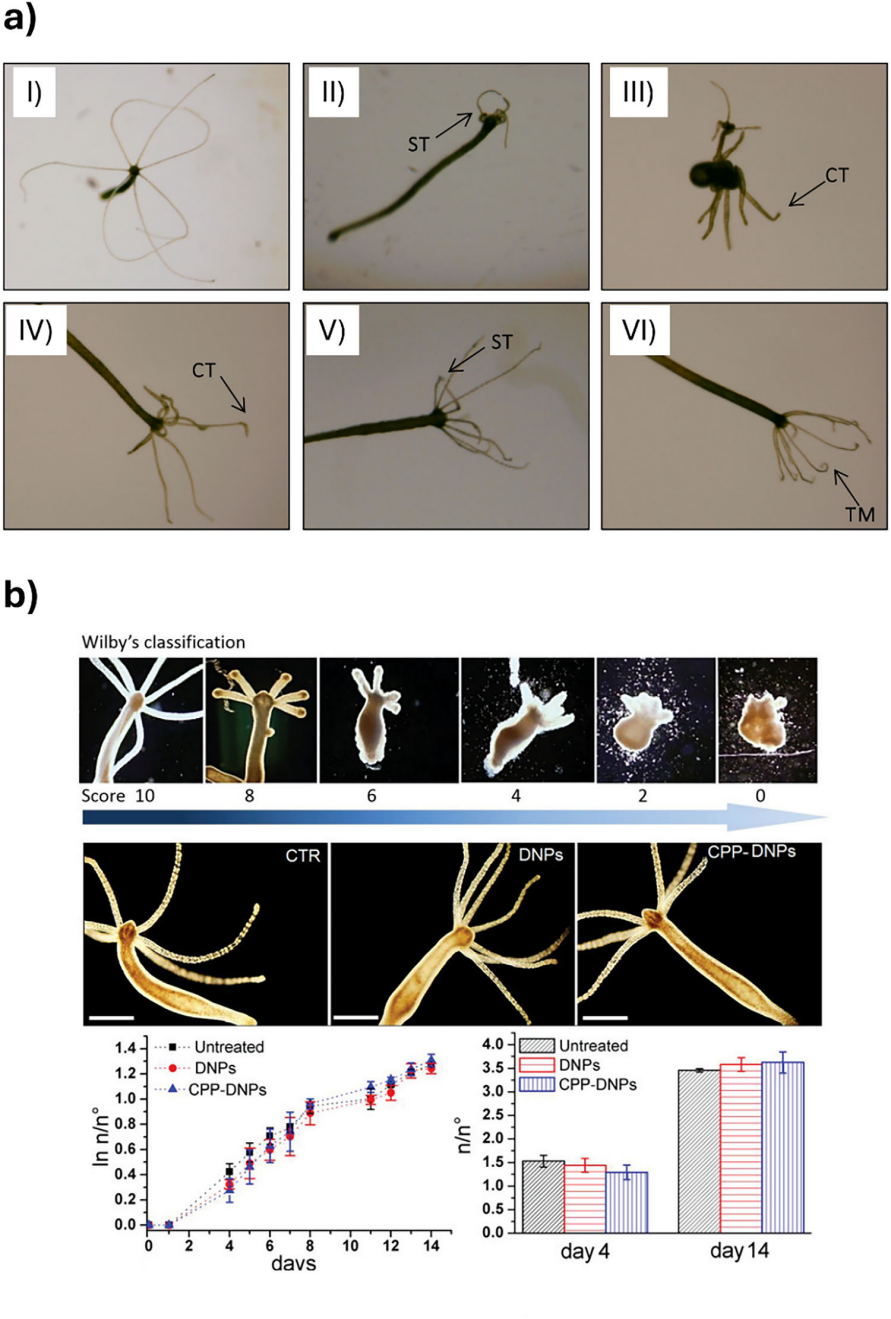
Overview of representative studies using *Hydra spp*. as an alternative model for testing NMs. (a) Representative malformations observed in *H. viridissima* polyps after 12 days of exposure to increasing concentrations of approximately 200‐nm polyhydroxybutyrate nanoparticles (PHB‐NPLs). (I) Control at day 12; (II) Control at day 10; (III) 0.001 mg PHB‐NPLs/L at day 6; (IV) 0.01 mg PHB‐NPLs/L at day 6; (V) 1 mg PHB‐NPLs/L at day 10; (VI) 1 mg PHB‐NPLs/L at day 6. Arrows indicate the malformations detected in each organism. CT: cubed tentacles; ST: shortened tentacles; TM: tentacle malformations characterized by curled tips [129]. (b) Analysis of the in vivo effects of hybrid diatomite nanovectors on H. vulgaris through morphological assessment, imaging of treated vs. control polyps, and evaluation of growth dynamics ratios over time [127]. Adapted with permission from [127, 129].

The freshwater cnidarian *Hydra spp*. has proven to be a powerful and sensitive model for evaluating the biological effects of ENMs, offering a unique bridge between in vitro cellular assays and vertebrate models in nanotoxicology. Representative examples of *Hydra spp*. used as an alternative for ecotoxicology investigations is presented in Figure 4. According to the reviewed studies, *Hydra vulgaris* and *Hydra viridissima* are the predominant species employed, each contributing distinct advantages to NP research.

*H. vulgaris*, used in studies involving DNA‐coated Au NPs [124], quantum rods [125, 126], and diatomite‐based silica carriers [127], is characterized by its transparent body and well‐mapped regenerative and developmental pathways, making it ideal for in vivo imaging and molecular tracking of NP uptake, distribution, and interepithelial migration. Conversely, *H. viridissima*, featured in studies on polyhydroxybutyrate (PHB) nanoplastics and Ag nanostructures, possesses symbiotic algae and exhibits heightened sensitivity to environmental stressors, particularly in assays assessing feeding behavior, reproduction, and long‐term ecological impacts [128, 129, 130]. This species’ green pigmentation and metabolic interactions with its symbionts may influence NP bioavailability and toxicity, as evidenced by altered feeding rates and reproductive depression upon PHB NP exposure [128]. Notably, co‐exposure studies in *H. viridissima* reveal that PHB NPs can modulate metal toxicity in a concentration‐ and metal‐specific manner, with both ameliorative and potentiating effects depending on the metal and NP dose, highlighting the species’ utility in mixture toxicity assessments [129]. Meanwhile, *H. vulgaris* enables high‐resolution tracking of NP‐cell interactions, with amino‐polyethylene glycol (PEG) quantum rods demonstrating charge‐dependent uptake mediated by annexin XII, and revealing novel intercellular trafficking dynamics during regeneration [125]. Diatomite‐based carriers functionalized with cell‐penetrating peptides showed efficient uptake and negligible toxicity in *H. vulgaris*, with no impact on morphology, apoptosis, or population growth, and only transient gene expression changes [127].

These interspecies differences underscore the importance of species selection in nanotoxicological studies: *H. vulgaris* excels in mechanistic and imaging‐based investigations, while *H. viridissima* offers ecological relevance and heightened sensitivity to sublethal stress. Collectively, the *Hydra* genus provides a multifaceted platform for probing NP biocompatibility, uptake mechanisms, and long‐term effects, with implications for human health and environmental safety.

#### 3.2.2 Bivalves

**FIGURE 5:**
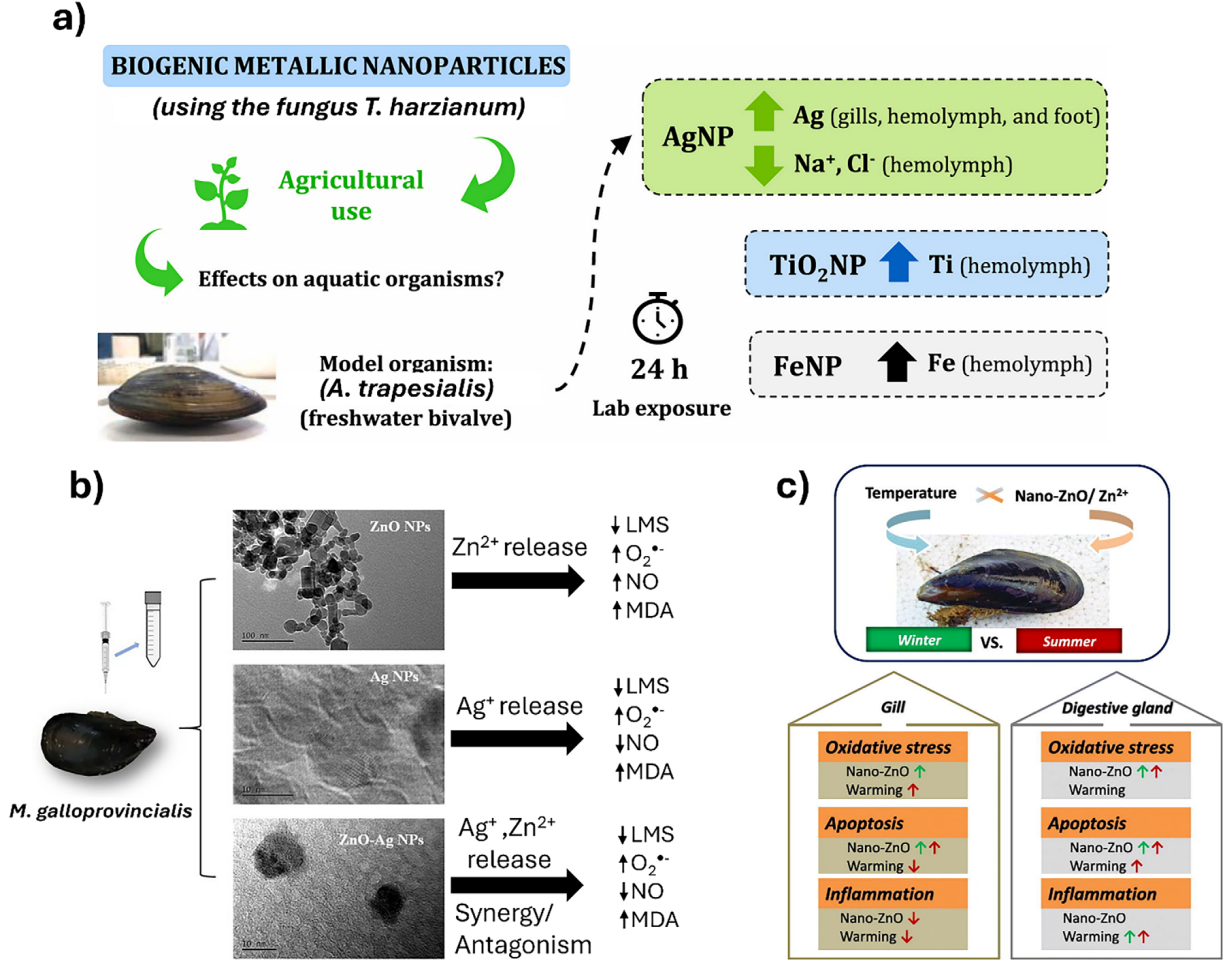
Overview of representative studies exploiting bivalve mollusks as alternative models for testing NMs. (a) Biocompatibility assessment of biogenic metallic NPs on the mussel A. trapesialis [137], (b) toxicity analysis of various nanostructures on *M. galloprovincialis* [139], (c) combined effects of temperature and ZnO NPs on *M. edulis* [142]. Adapted with permission from [137, 139, 142].

Studies investigating the effects of NPs and NMs on aquatic invertebrates, including bivalves and freshwater mussels, reveal a broad spectrum of toxicological responses that vary according to NP type, size, concentration, and exposure duration: an overview of representative studies using bivalves as an alternative model for ecotoxicology studies is presented in Figure 5.

TiO_2_ NPs have been extensively examined, and even short‐term exposure (7 days) to small‐sized TiO_2_ NPs (5–15 nm) has been shown to cause immunosuppression, metabolic disruption, oxidative stress, and severe histopathological damage in *Lamellidens marginalis* [131]. Similar effects, including marked histological alterations, immune modulation, DNA damage, and gonadal impairment after prolonged exposure (28 days), have been reported in *Caelatura nilotica* [131, 132]. The use of the chelating agent dimercaptosuccinic acid (DMSA) has been noted to mitigate some of these effects, underscoring the importance of protective factors in toxicity outcomes.

Compared with other NMs, ZnO NPs and ZnO/alginate nanocomposites induce pronounced oxidative stress and tissue‐level damage in *Coelatura aegyptiaca*, particularly in the composite forms, suggesting that hybrid formulations can enhance toxicity [133]. Similarly, Au‐doped nanoplastics and PS nanoplastics bioaccumulate in bivalves and zooplankton, producing measurable oxidative damage and DNA strand breaks. Despite low overall mortality, these responses represent sublethal but ecologically significant effects [134, 135].

Comparative studies involving TiO_2_, Al_2_O_3_, and CuO NPs indicate tissue‐specific accumulation, with metal concentrations typically highest in gills, but without observable mortality, implying differences in uptake efficiency and toxicity thresholds among NP types [136]. Biogenic TiO_2_ NPs (∼230 nm) generally exert negligible physiological effects in *Anodontites trapesialis*, whereas Ag NPs induce ionoregulatory imbalance, highlighting the influence of synthesis method and particle size on toxicity [137]. Meta‐analytical assessments identify oxidative stress, ROS generation, and enzyme disruption as consistent mechanistic pathways across studies, and emphasize the need for standardized methodologies and adverse outcome pathways (AOP)‐based frameworks to improve the predictability of NP effects in freshwater invertebrates [138].

Marine bivalves have emerged as pivotal bridging models for assessing the toxicological effects of NPs and NMs on human health, offering a unique combination of ecological relevance, physiological sensitivity, and molecular comparability to higher organisms. Across multiple studies, species such as *Mytilus galloprovincialis*, *Mytilus edulis*, *Ruditapes philippinarum*, and *Tegillarca granosa* have been exposed to a diverse array of NPs—including metal‐ and metal oxide‐based (ZnO, Ag, Au‐ZnO, TiO_2_, antimony‐doped tin oxide ‐ATO‐), and polymeric (PS nanoplastics)—to evaluate their sub‐lethal and chronic effects. A consistent finding across these investigations is the induction of oxidative stress, genotoxicity, and immune modulation, which are hallmark responses also observed in mammalian systems, thereby reinforcing the translational value of bivalves in human health risk assessment [139, 140, 141, 142, 143, 144, 145].

Despite these shared mechanisms, substantial variations exist in NP behavior, toxicity pathways, and tissue‐specific responses. Ag NPs are generally more cytotoxic to mussel hemocytes than ZnO or ZnO–Ag composites, with lysosomal membrane destabilization and lipid peroxidation identified as major endpoints [139]. In contrast, PS nanoplastics elicit chronic genotoxic and neurotoxic effects, displaying tissue‐specific accumulation patterns with maximal uptake in gills and digestive glands and pronounced accumulation in gonads—implicating potential reproductive impairment [140, 141]. These findings were further supported by modeling approaches correlating in vitro hemocyte responses with in vivo tissue damage, demonstrating strong predictive power for apoptosis and DNA damage [146].

A novel dimension was introduced by examining the role of seasonal temperature variation in modulating ZnO NP toxicity, showing that higher summer temperatures exacerbate oxidative and apoptotic responses, while winter conditions tend to mitigate toxicity, underscoring the importance of environmental context in NP risk assessment [142]. Similarly, hypoxia amplifies metabolic disruption and mortality induced by ZnO NPs, with limited recovery upon reoxygenation [143]. Comparisons of different Ag forms show that ionic Ag^+^ species (AgNO_3_) are more persistent and more disruptive to byssal adhesion than nanoparticulate forms, despite similar acute mortality rates [144].

Combined exposures further illustrate the complexity of NM interactions. Co‐exposure to Au–ZnO NPs and phosphonate compounds results in antagonistic interactions that mitigate oxidative and neurotoxic effects [147]. In contrast, mixtures of nanoplastics and nano‐TiO_2_ alter the gill‐associated microbiome of *Laternula elliptica*, promoting opportunistic taxa and perturbing metabolic functions, indicative of synergistic toxicity [148]. Additional evidence shows that particle size, crystal structure, and environmental co‐stressors such as ocean acidification and warming markedly influence TiO_2_ NP outcomes, with larger particles generally exhibiting higher toxicity [149].

Abiotic factors also shape metabolic and immune responses. Exposure to various NPs suppresses key metabolic pathways in *Tegillarca granosa*, while changes in salinity modulate Ag NP toxicity in *Saccostrea cucullata*, with lower salinity enhancing immunosuppression and oxidative stress [145, 150]. These findings highlight the importance of abiotic factors in shaping NP toxicity profiles. Finally, ATO NPs display low acute toxicity but measurable bioaccumulation in *M. galloprovincialis*, pointing to potential long‐term risks through seafood consumption [151]. Although no structural damage is typically observed, such findings emphasize the need for chronic exposure assessments, consistent with broader concerns about the latent effects of nanopollutants.

In summary, while all studies converge on the utility of marine bivalves as sentinel organisms capable of revealing conserved toxicity mechanisms relevant to human health, they diverge in their emphasis on NP type, environmental context, exposure duration, and biological endpoints. This diversity of approaches enriches our understanding of NP behavior and toxicity, and collectively supports the integration of bivalve‐based models into human health risk frameworks.

#### 3.2.3 Planarians

**FIGURE 6:**
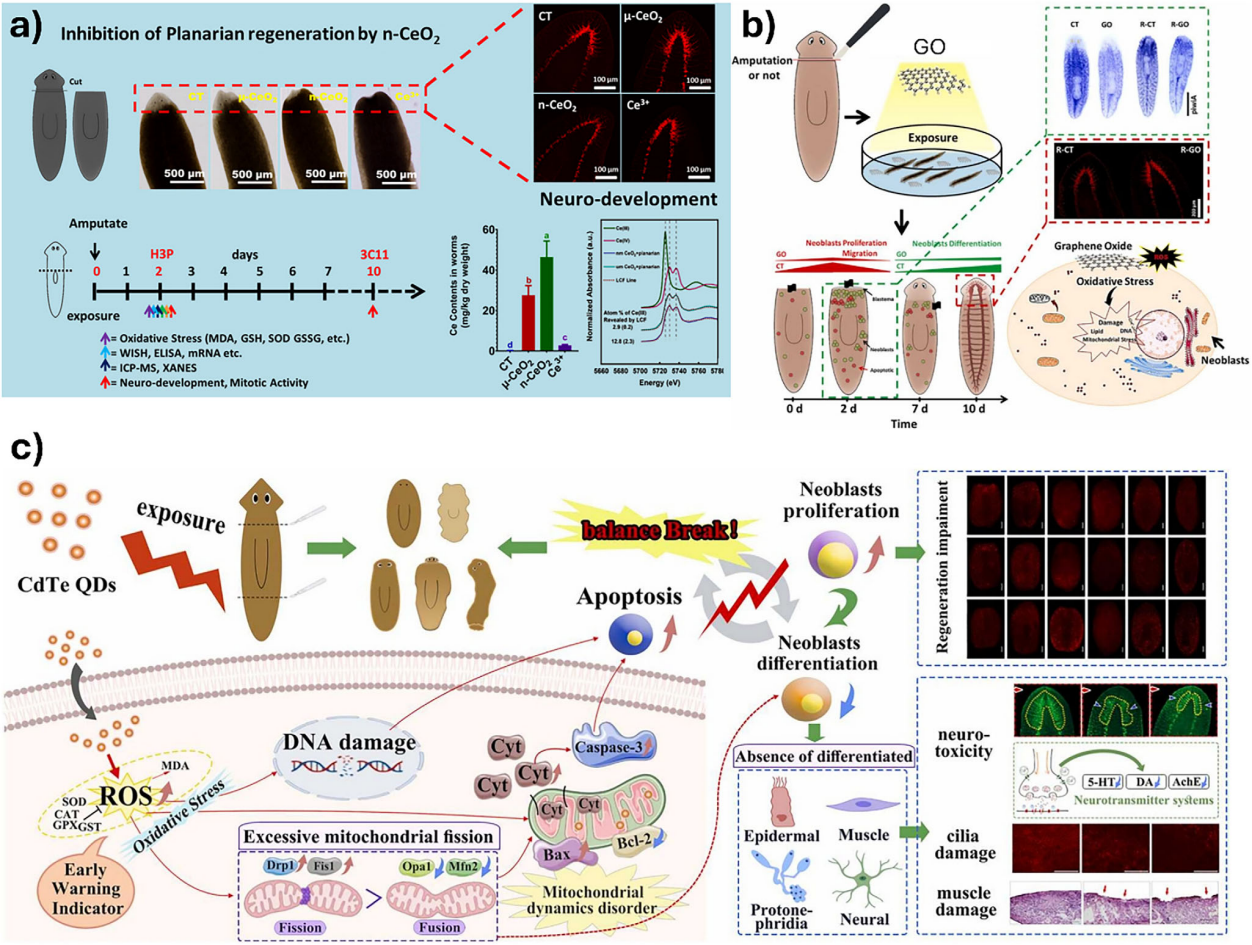
Overview of representative studies using planarians as alternative models for testing NMs. (a) Testing of CeO_2_ nanoparticles [334], (b) testing of GO [157], (c) testing of CdTe QGs [159]. Adapted with permission from [157, 159, 334].

Planarians have emerged as a valuable model for evaluating the ecotoxicological impacts of ENMs, particularly due to their regenerative capacity, sensitivity to environmental stressors, and well‐characterized stem cell biology. A variety of studies have explored how different types of NPs affect physiological, molecular, and behavioral endpoints in different planarian species, revealing both common mechanisms and material‐specific responses. Representative examples of these studies are presented in Figure 6.

Ag NPs are among the most extensively studied and are reported to inhibit regeneration in *Girardia tigrina*, specifically by reducing blastema area following decapitation, consistent with impaired cell proliferation [152]. In *Schmidtea mediterranea*, Ag NPs disrupt bacterial community composition in adults, but show weaker effects during regeneration, likely reflecting the dynamic microbiome shifts accompanying tissue remodeling [153]. These findings underscore how physiological state modulates NP sensitivity. Investigations with Ag_2_S NPs indicate substantial bioaccumulation in *G. tigrina* without evidence of biomagnification through trophic transfer, particularly following dietary exposure [154].

While Ag‐based materials generally impair regeneration and disrupt physiological homeostasis, CeO_2_ NPs can exhibit either protective or modulatory effects. Under low‐dose radiation or altered gravity, CeO_2_ NPs preserve stem cell activity and reduce apoptosis in planarians, effects attributed to the antioxidant properties of the NPs [155, 156]. In contrast, CeO_2_ NPs, unlike their bulk counterparts, can disrupt regeneration, stem cell proliferation, and neurodevelopment in *Dugesia japonica*, largely via partial biotransformation to toxic Ce^3^^+^ ions [157]. Thus, Ce‐based NMs may act either protectively or toxically depending on their physicochemical form and biotransformation in vivo. Both CeO_2_ and CeF_3_ NPs also confer radioprotective effects in *S. mediterranea*, and are associated with upregulation of regeneration‐related genes, suggesting gene‐level modulation as a complementary mechanism [158].

Other ENMs display pronounced toxicity in planarians. Cadmium telluride QDs (CdTe QDs) induce extensive phenotypic and molecular damage *in Dugesia constrictiva*, including disrupted stem cell differentiation, neurotoxicity, oxidative stress, and apoptosis, with effects mediated by mitochondrial dysfunction and ROS overproduction [159]. Carbon QDs (CQDs) instead impair central nervous system regeneration in *D. japonica* by disturbing Hedgehog pathway signaling, reducing expression of neural markers, and ultimately leading to lethal morphological defects [160].

Overall, these studies confirm that NP toxicity in planarians is highly context‐dependent, influenced by material type, exposure route, particle size, ionic dissolution, regeneration state, and molecular pathways engaged. While some ENMs, such as nanoceria, can support regeneration under stress, others, like CdTe QDs and carbon QDs, are clearly deleterious. This variability highlights the need for comprehensive, comparative assessments using multiple endpoints in diverse planarian species to better inform environmental nanotoxicology and NP risk assessment.

#### 3.2.4 Zebrafish

**FIGURE 7:**
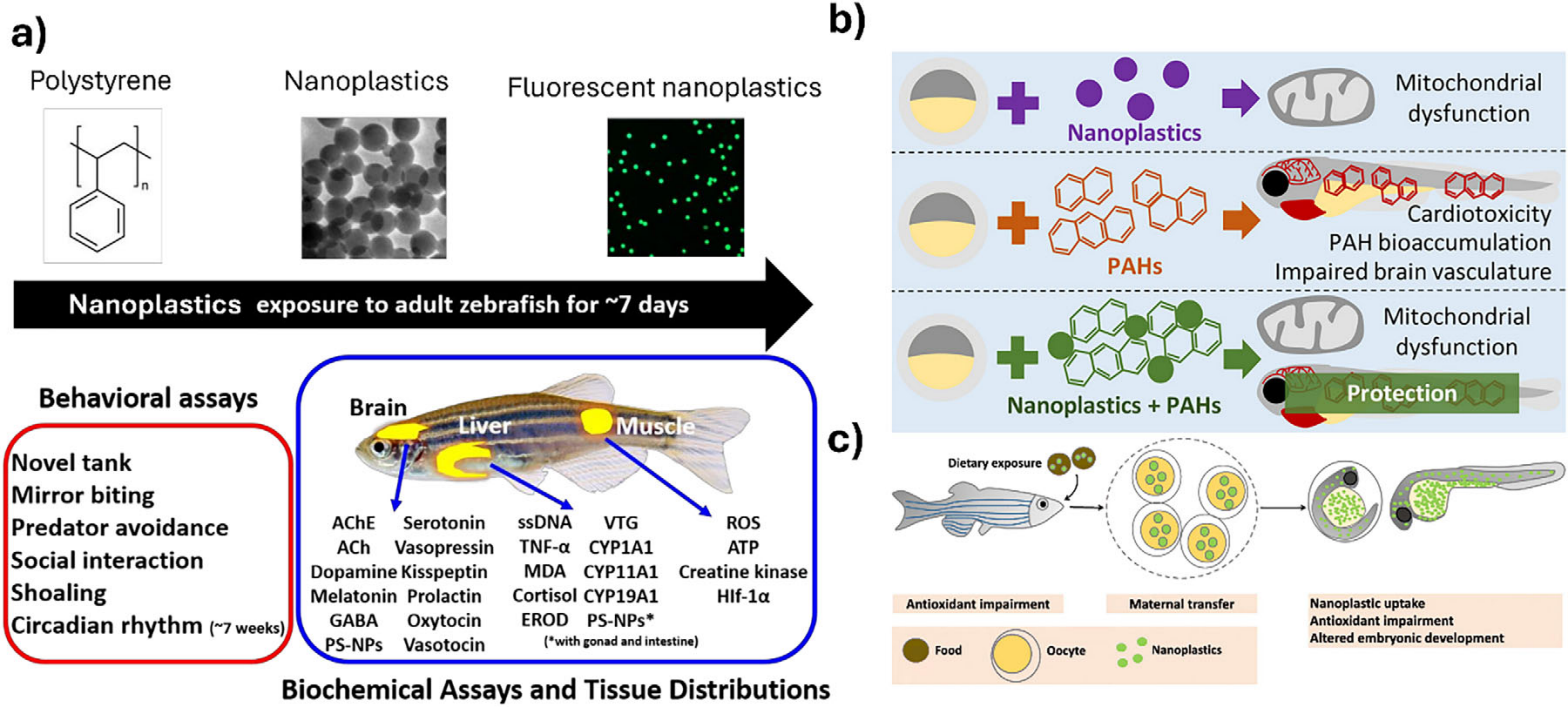
Overview of representative studies using zebrafish as an alternative model for testing NMs. (a,b) Testing of nanoplastics [113, 120], (c) maternal transfer of nanoplastics across generations in zebrafish [116]. Adapted with permission from [113, 116, 120].

Zebrafish have emerged as a robust and versatile vertebrate model for evaluating the biological effects of ENMs, providing a valuable bridge between in vitro assays and mammalian systems in nanotoxicology, as presented in Figure 7. Their high genetic homology to humans, rapid embryonic development, optical transparency, and amenability to high‐throughput screening make them particularly well‐suited for assessing developmental, physiological, and molecular responses to NMs. Across the reviewed studies, zebrafish were consistently employed to investigate the biodistribution, toxicity, and overall biological impacts of various ENMs, including metal‐based NPs (e.g., TiO_2_, ZnO, Ag, Au), silica particles, and PS nanoplastics.

Common findings across these studies include the capacity of NPs to penetrate biological barriers such as the chorion [111, 119], induce oxidative stress and apoptosis [115, 161], and disrupt physiological processes such as heart rate and microbial homeostasis [115, 116]. Phototoxicity has been observed to be life‐stage dependent, with yolk‐sac larvae exhibiting greater sensitivity to TiO_2_ NPs under simulated solar radiation, emphasizing the influence of developmental timing on toxicity outcomes [162]. Modifications of NP surface chemistry, such as those applied to Au nanorods, significantly modulate toxicity profiles, underscoring the importance of physicochemical properties in determining biological interactions [161].

Beyond acute toxicity, transgenerational effects have been documented following dietary exposure to PS nanoplastics in adult zebrafish, leading to maternal transfer of particles to offspring and concurrent antioxidant impairment in both generations [116]. Beyond acute toxicity, transgenerational effects have been documented following dietary exposure to PS nanoplastics in adult zebrafish, leading to maternal transfer of particles to offspring and concurrent antioxidant impairment in both generations [119].

Collectively, these studies underscore the zebrafish model's sensitivity to NP‐induced perturbations and its capacity to reveal nuanced biological responses across molecular, cellular, and organismal levels. The convergence of findings —particularly regarding oxidative stress, developmental disruption, and bioaccumulation—, reinforces the relevance of zebrafish in predicting potential human health risks associated with NM exposure. At the same time, differences in exposure routes, NP types, and life‐stage susceptibility highlight the need for standardized protocols and comprehensive multi‐endpoint approaches. As nanotechnology continues to expand, the zebrafish model stands as a critical tool for elucidating the complex interactions between ENMs and biological systems, informing both environmental safety and human health risk assessments.

#### 3.2.5 Caenorhabditis Elegans

**FIGURE 8:**
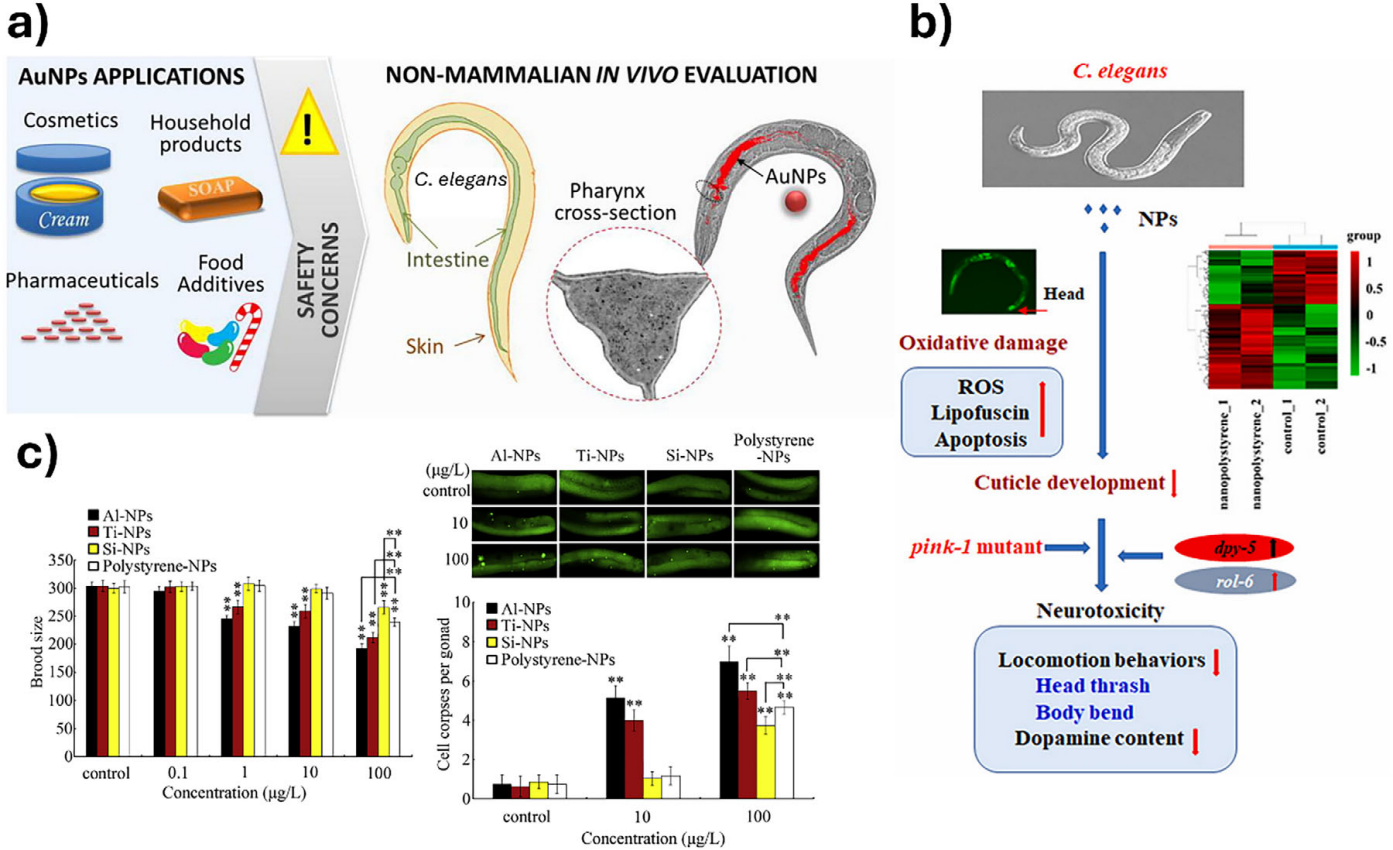
Overview of representative studies using *C. elegans* as an alternative model for testing NMs. (a) Testing of gold NPs [167], (b) comparison of the toxic effects of PS NPs and three metal oxide NPs on brood size reduction (left) and germline apoptosis induction (right) [166], (c) analysis of PS NPs toxicity [165]. Adapted with permission from [165, 166, 167].

*C. elegans* has emerged as a robust and versatile in vivo model for evaluating the biological effects of ENMs, offering a valuable bridge between in vitro assays and mammalian models in nanotoxicology. Across diverse studies, *C. elegans* has demonstrated sensitivity to a wide range of NPs, including QDs [163], superparamagnetic iron oxide NPs (SPIONS) [164], PS nanoplastics [165, 166], Au NPs [167], and Ag NPs [168, 169, 170]. Representative studies are presented in Figure 8. These investigations consistently highlight the organism's capacity to reveal sublethal endpoints such as reproductive impairment, neurodevelopmental toxicity, oxidative stress, and mitochondrial dysfunction.

QDs induce egg‐laying defects and reduced lifespan via motor neuron disruption [163], while PS NPs alter locomotion and dopamine levels through oxidative damage and gene regulation [165]. Similarly, bovine serum albumin (BSA)‐coated SPIONs reduced acute toxicity compared to citrate‐coated variants, underscoring the role of surface chemistry in biodistribution and uptake [164]. Nanopolystyrene displays intermediate toxicity compared with metal oxide NPs, with oxidative stress and activation of the mitochondrial unfolded protein response (mtUPR) identified as key mechanisms [166].

Au NPs, although largely confined to the intestinal lumen, exhibit size‐dependent toxicity and aggregation behavior, with smaller particles exerting greater effects on reproduction [167]. Ag NPs, among the most extensively investigated materials, demonstrate both ion‐mediated and particle‐specific toxicity, with sulfidation markedly reducing bioavailability and adverse effects [168]. Evidence also indicates intracellular uptake and transgenerational transfer of Ag NPs, with metallothionein‐deficient strains showing increased sensitivity [169]. Other findings reveal that surface contact alone can trigger dermal toxicity, as epidermal damage and edema occur even in the absence of confirmed internalization [170].

Overall, these studies underscore the relevance of *C. elegans* in capturing complex nano‐bio interactions, including uptake pathways, genetic responses, and systemic effects, while offering logistical advantages such as genetic tractability, transparency, and ethical acceptability. The convergence of findings across NP types and functionalization, alongside mechanistic insights into oxidative stress, neurotoxicity, and reproductive disruption, supports the utility of *C. elegans* as a predictive and scalable model for assessing NP safety and informing about human health risk assessments.

#### 3.2.6 Bombyx Mori

**FIGURE 9:**
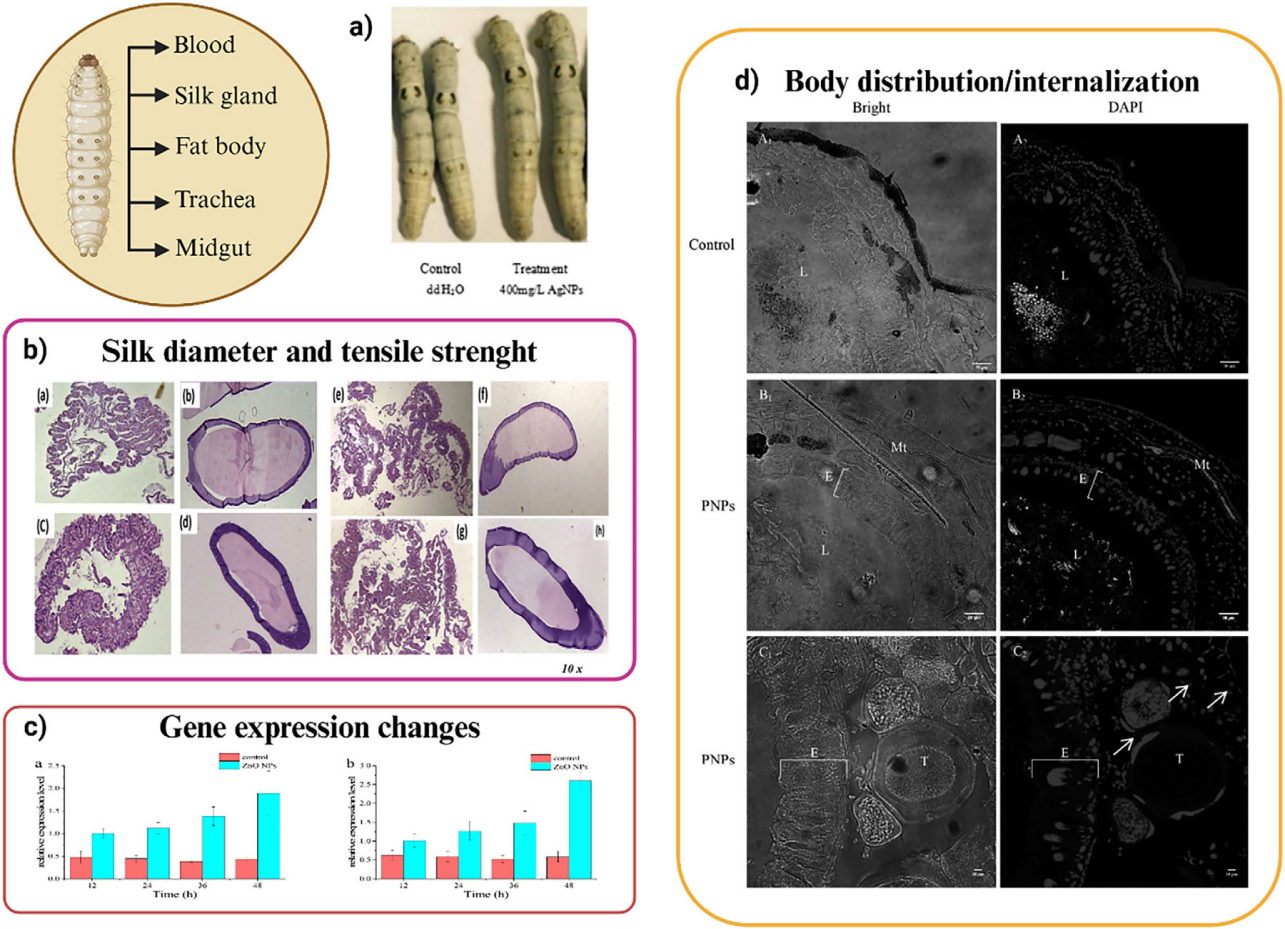
Overview of representative studies using *B. mori* as an alternative model for testing NMs. (a) Morphological abnormalities of silkworms after feeding with AgNPs [335] (b) histopathology images of silkworm tissues after treatment with TiO_2_ NPs [173], (c) Expression patterns of Dronc (a) and Caspase (b) in midguts after treatment with ZnO NPs [171], (d) detection of the internalization of polystyrene nanoparticles [175]. Adapted with permission from [171, 173, 175, 335].

The silkworm *Bombyx mori* is increasingly recognized as a robust “bridging” invertebrate for assessing the environmental hazards of emerging contaminants, because it combines low ethical burden and low cost with a whole‐organism complexity (digestive tract, fat body, haemolymph/immune cells, excretory system) that enables biodistribution, toxicokinetics, and organ‐specific readouts not accessible in simpler models; notably, its sequenced genome also supports mechanistic interpretation (e.g., gene‐expression changes and adverse outcome pathway building) relevant to nanosafety [171]. Representative studies are presented in Figure 9.

In nanoparticle ecotoxicology, silkworms allow both environmentally realistic oral exposure (dietary delivery via mulberry leaves) and controlled acute dosing (e.g., injection), with sensitive endpoints spanning bioaccumulation, oxidative stress, immune perturbation, and apoptosis signaling: ZnO NPs showed tissue biodistribution with prominent midgut burden and activation of antioxidant enzymes alongside apoptosis‐related transcriptional changes [171], while AgNPs altered haemocyte profiles and antioxidant/catalase‐related responses after dietary exposure [172]. Metal‐oxide particles are also informative for chronic, diet‐based hazard screening, as TiO_2_ NPs produced dose‐ and size‐dependent effects on growth traits and accumulated in the midgut, with measurable downstream impacts at tissue and molecular levels [173], and low‐dose TiO_2_ NPs modified the intestinal microbiota composition in *B. mori*, illustrating how nanoparticles can indirectly affect host physiology via the gut ecosystem [174].

Importantly for microplastic/nanoplastic research, ingestion studies have demonstrated that polystyrene nanoplastics can be detected in larval compartments and are associated with behavioral disturbances, supporting *B. mori* as a terrestrial‐relevant model for plastic particle uptake and organism‐level outcomes [175]. Overall, together with its well‐established husbandry and extensive biomarker repertoire, these data support the silkworm as a practical alternative model to evaluate the dual (beneficial vs. adverse) impacts of nanotechnologies across health‐ and environment‐relevant endpoints, while acknowledging that inter‐phyla differences still limit direct quantitative extrapolation to mammals [96].

#### 3.2.7 Drosophila Melanogaster

**FIGURE 10:**
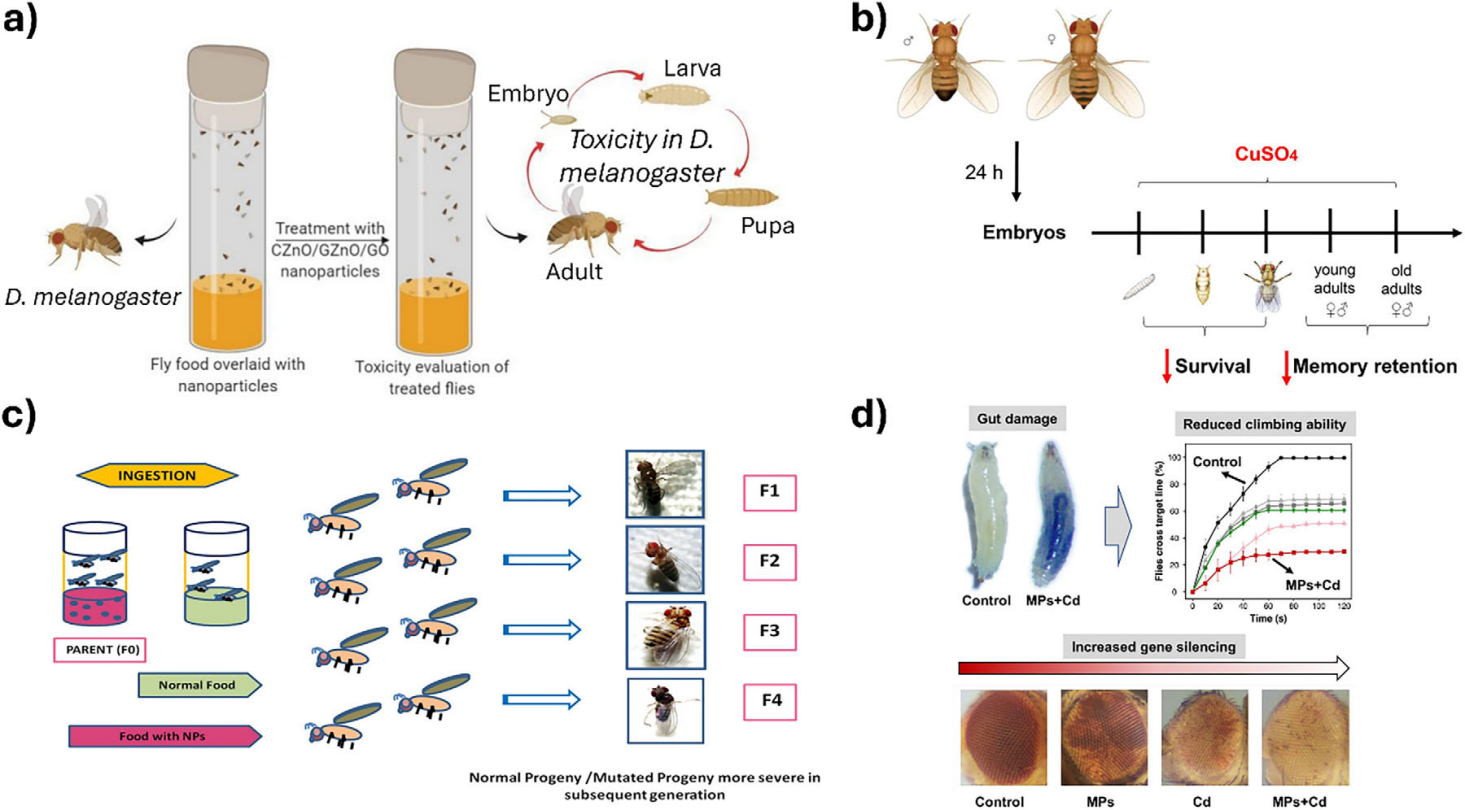
Overview of representative studies using *D. melanogaster* as an alternative model for testing NMs. (a) Testing of GO and ZnO NPs [176], (b) effects of CuSO_4_ exposure [180], (c) testing of ZnO NPs [178], d) testing of microplastics [182]. Adapted with permission from [176, 178, 180, 182].

*D. melanogaster* is increasingly recognized as a powerful and ethically sound in vivo system for investigating the biological and toxicological impacts of ENMs, serving as a critical intermediary between cell‐based assays and complex mammalian models in the field of nanotoxicology (Figure 10). Numerous studies have shown that *D. melanogaster* is highly responsive to a diverse array of NPs—such as ZnO, graphene oxide (GO), Au NPs, Co, Cu, Ag, S, and microplastics (MPs)—eliciting a wide spectrum of biological effects, including cytotoxicity, genotoxicity, neurotoxicity, oxidative stress, and morphological anomalies.

Comparative analyses of GO and ZnO NPs synthesized via different methods reveal distinct cytotoxic and neurotoxic profiles, with chemically synthesized ZnO exhibiting greater cytotoxicity and green‐synthesized ZnO showing enhanced neurotoxicity [176]. ZnO NPs can cross the intestinal barrier without inducing overt genotoxicity or oxidative stress, although alterations in the expression of stress‐related genes such as *Hsp70* and *p53* have been reported [177]. Under chronic exposure, ZnO NPs trigger oxidative stress, DNA fragmentation, and heritable phenotypic abnormalities, suggesting genotoxic potential upon prolonged contact [178]. Co NPs induce somatic recombination in wing tissues, producing genotoxicity patterns distinct from those caused by ionic cobalt and underscoring the importance of NP form and cellular uptake [179]. Copper exposure impairs associative learning and memory, with sex‐dependent susceptibility, further suggesting *D. melanogaster*’s utility in neurobehavioral toxicology [180].

Au NPs exhibit concentration‐dependent but size‐independent toxicity, influencing lifespan, fertility, ROS levels, and gene expression [181]. MP exposure exacerbates cadmium toxicity, leading to gut damage, locomotor dysfunction, and epigenetic silencing through position‐effect variegation [182]. Green‐synthesized Ag and sulfur NPs show insecticidal potential, inducing significant larval and pupal mortality and egg deterrence, particularly with Ag NPs derived from plant‐based extracts such as olive and mulberry [183].

Overall, these studies underscore *D. melanogaster*’s versatility in modeling NP‐induced effects, capturing both acute and chronic responses, and enabling mechanistic insights into oxidative stress, genotoxicity, and neurotoxicity. Differences in synthesis methods, exposure duration, NP composition, and endpoints highlight the need for standardized protocols, yet the convergence of findings across diverse ENMs affirms *D. melanogaster*’s translational relevance for human health risk assessment.

### 3.3 From Ecotoxicology to Nanomedicine

Overall, ecotoxicological investigations using alternative models have provided crucial insights into the environmental behavior and biological impact of ENMs across phylogenetically diverse species. Despite their simplicity, these organisms capture conserved toxicological mechanisms such as oxidative stress, immune modulation, and metabolic disruption—processes that are also central to NP interactions in higher vertebrates. By revealing these shared molecular and physiological responses, ecotoxicological studies not only inform environmental risk assessment but also strengthen the translational bridge between environmental and biomedical nanosciences. Building on this integrative framework, the following section examines how these same models can be leveraged to assess the therapeutic performance, biocompatibility, and safety of NMs in preclinical nanomedicine.

## 4 Alternative Models for Developing and Testing New Nanomedicines

Bridging models are well‐suited to evaluate both toxicity and therapeutic performance of NMs. To complement the ecotoxicology section, this part focuses on translational models used to test nanotherapeutics for human health.

### 4.1 Nanotoxicity Assessment and Biocompatibility of Nanotherapeutics in Preclinical Bridging Models

**FIGURE 11:**
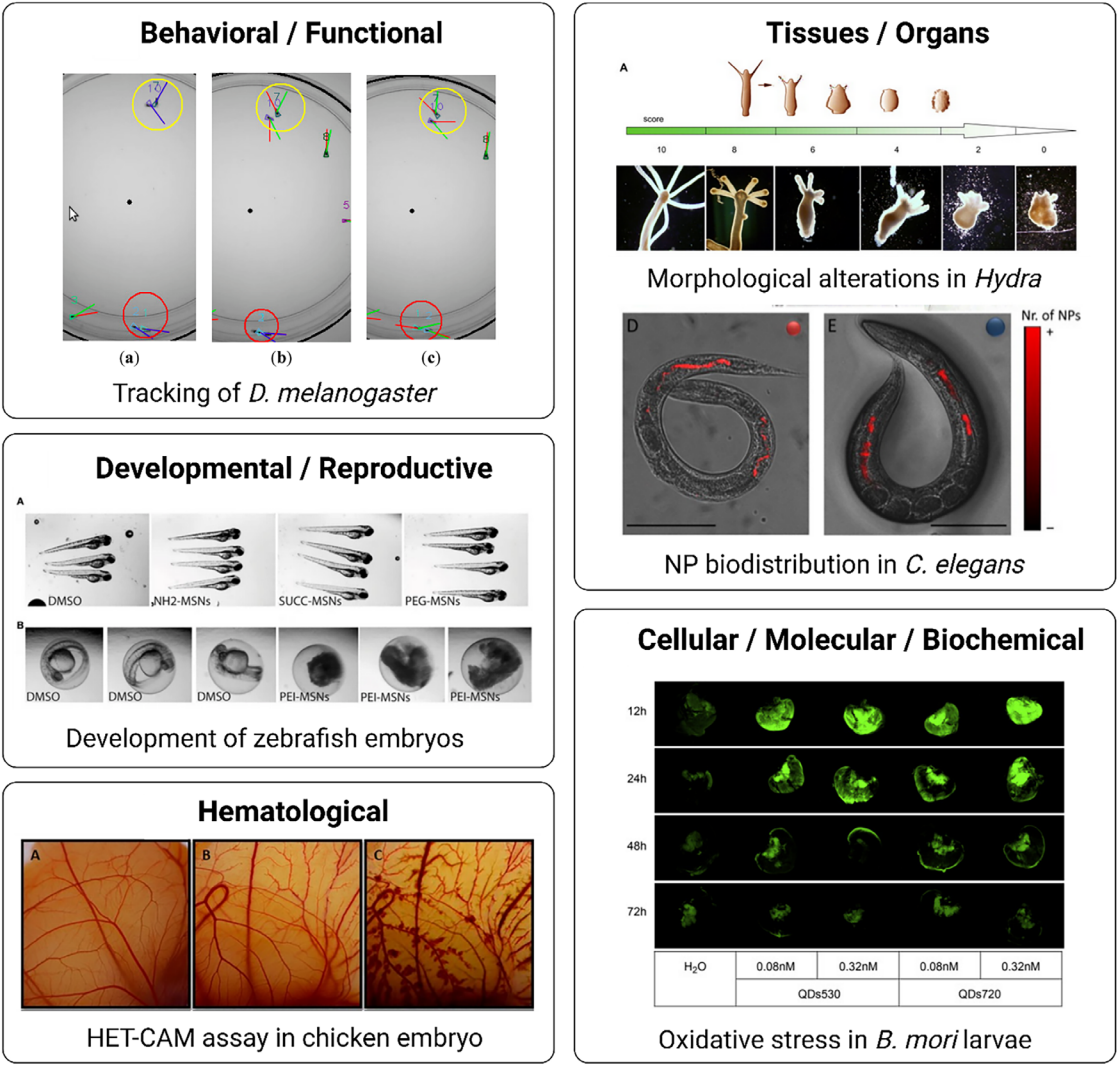
Representative examples of the use of bridging models for preclinical nanotherapeutic evaluation at different biological levels, from survival and behavior to organ pathology, hematology, and molecular mechanisms. Adapted with permission from [167, 185, 188, 194, 208, 250].

Nanotherapeutics show promise in oncology and targeted delivery, but preclinical toxicity and biocompatibility remain essential. Beyond conventional in vitro and in vivo assays, bridging models improve predictive value while reducing reliance on larger mammals. Among them, the CAM model remains the gold standard [106, 109], although more recent interest has extended to invertebrates such as *H. vulgaris*, zebrafish, *C. elegans*, *D. melanogaster*, and *B. mori*. They enable multi‐scale readouts—from survival and behavior to organ pathology, hematology, and molecular mechanisms (Table 2, Figure 11).

**TABLE 2:** Experimental bridging models and their evaluation criteria for nanotherapeutic studies.

Biological level	Evaluation Criteria	Model	References
Behavioral / Functional	Behavior	*C. elegans*	[187]
		*D. melanogaster*	[192, 193]
		Zebrafish	[189]
Developmental and Reproductive	Lethality, development, reproduction, and weight	CAM	[195, 196, 197, 198, 199, 200, 201, 202, 203, 204, 205, 206]
		*H. vulgaris*	[127, 185, 186]
		*D. melanogaster*	[177, 192, 193]
		*C. elegans*	[164, 167, 184, 187]
		Zebrafish	[161, 188, 189, 190, 191]
		*B. mori*	[194]
Tissue / Organ	Morphological changes, organ weight, cardiovascular function, biodistribution, and regeneration	CAM	[195, 202, 203, 206, 222, 224]
		*H. vulgaris*	[127, 185, 186]
		*D. melanogaster*	[177, 192]
		Zebrafish	[161, 188, 189, 191]
Hematological	Vascular damage/changes, blood cells analysis, blood serum analysis	CAM	[109, 196, 198, 199, 201, 202, 203, 205, 207, 208, 209, 210, 211, 212, 213, 214, 215, 216, 217, 218, 219, 220, 221, 222, 223, 224, 225, 226, 227]
		*B. mori*	[194]
Cellular / Molecular / Biochemical	Cell proliferation, oxidative stress, gene expression, biochemical and molecular responses, metabolism, and genotoxicity	CAM	[202, 203, 222]
		*D. melanogaster*	[177, 192, 336]
		*C. elegans*	[167, 184, 231]
		*H. vulgaris*	[127, 185, 186]
		*B. mori*	[194]
		Zebrafish	[161]

Routes of exposure vary by model. Aquatic species (*H. vulgaris*, zebrafish, *C. elegans*) are typically exposed via the medium [127, 161, 164, 167, 184, 185, 186, 187, 188, 189, 190, 191], whereas *D. melanogaster* via diet [177, 192, 193]. In *B. mori*, the only reported nanotoxicity study involved intravascular injection of QDs [194]. In the CAM model, routes of exposure are more diverse, including deposition onto the CAM [195, 196, 197, 198], intravascular injection [196, 199, 200], mesodermal injection [201], injection into the albumen [195, 202, 203, 204, 205], and even direct injection into the heart [206]. The choice depends on both model feasibility and the intended administration route of the nanotherapy under development, for instance, topical treatments (eyes or skin) are usually tested by CAM surface application [207, 208, 209, 210, 211, 212, 213, 214, 215, 216, 217, 218, 219, 220, 221], while anticancer drug delivery systems are assessed via intravascular administration [200, 222, 223].

The most common and straightforward outcome in nanotoxicity studies consists of evaluating the survival of the whole organism following NP exposure. All models described in this Section have been employed to assess lethality from various nanotherapies. Reproductive performance provides important insights into nanotoxicity in non‐embryonic models such as *H. vulgaris* [185, 186] and *C. elegans* [164, 167, 184, 187], often reflecting potential genotoxic effects, such as downregulation of genes involved in reproduction [184]. Developmental and growth alterations (e.g., delayed hatching, reduced growth rate, or changes in body weight) have also been reported across several models [127, 177, 187, 191, 192, 193, 194, 195, 197, 202, 203, 204, 205, 222, 224]. For example, negatively charged CeO_2_ NPs delayed *D. melanogaster* larval development by one week, likely due to NP accumulation in lysosomes leading to cell death [193]. *Hydra spp*. is particularly suitable for studying the impact of NPs on regenerative processes, highlighting impaired cell division and cytotoxic effects [185, 186]. Behavioral and functional assays in *C. elegans* [187], *D. melanogaster* [191, 193], and zebrafish [189] further confirm toxicity outcomes: these analyses are described extensively in the previous part concerning ecotoxicology. For instance, hydroxyapatite NPs used for bone regeneration induced defective walking behavior in *D. melanogaster* [192], while chitosan NPs caused hyperactivity in zebrafish larvae by altering motor neurons and muscle structure [189].

At tissue and organ level, changes in organ weight, structure, and morphology are also considered indicators of nanotoxicity. For example, *in ovo* studies have evaluated the non‐teratogenic effects of carbon NPs and drug‐loaded polymeric NPs [202, 203, 222, 224], while *H. vulgaris*‐based assays have been used to assess morphological alterations [127, 185, 186]. Notably, after exposure to nanomolar doses of CdTe QDs, *H. vulgaris* death was preceded by progressive morphological alterations [185]. Similar QD‐induced alterations were also demonstrated in *D. melanogaster* [177], whereas hydroxyapatite NPs caused defects in wings, eyes, and thorax [192]. Reports involving *C. elegans* are fewer in the context of nanotherapies, in contrast to the numerous ecotoxicological studies described earlier. Organs can also be recovered to analyze ultrastructure, such as the brain [202], or to study biodistribution. For instance, *in ovo* experiments compared iron NP accumulation in the liver and kidney [195], while fluorescent‐labelled NPs allowed visualization of NP diffusion throughout the *H. vulgaris* body [125].

The *in ovo* model is particularly valuable for investigating the vascular effects of NPs—including vascular damage, angiogenesis, and hemorrhage—because of the accessibility of the embryo's circulatory system in the extra‐embryonic membrane [109]. One of the simplest and most widely used assays is the hen's egg test (HET)‐CAM test, which calculates the irritation score (IS) for assessing the irritant potential of substances applied to skin or eyes. The IS is based on three criteria—coagulation, hemorrhage, and vessel lysis—and ranges from 0 (non‐irritant) to 10 (severe irritation). This method has been extensively applied to NP evaluation, particularly for polymeric and lipid NPs intended for drug delivery through the eyes, skin, and nails: [207, 208, 209, 210, 211, 212, 213, 214, 215, 216, 217, 218, 219, 220, 221] NPs are applied directly onto the CAM surface, enabling rapid toxicological assessment. The IS test has also been performed as a complementary toxicological study for drug‐loaded polymer NPs designed for oral or intravenous administration in cancer and inflammatory disease therapy [223, 225], and has even been used to compare different magnetic iron oxide NPs for targeted anticancer drug delivery [226]. Beyond IS scoring, direct evaluation of the effect on vasculature has included inflammation, density of branched vessels, vascularization of the heart, and neoangiogenesis [196, 224, 227]. The *in ovo* model also enables blood sampling for blood‐cell morphology evaluation, including size, nuclear size, shape, and color, and presence/absence of cellular inclusions after exposure to carbon NPs, for instance [203]. Another example comes from *B. mori*, which has been used to study hematopoiesis. Following QD exposure, the proportion of abnormal hemocytes increased in a dose‐dependent manner, accompanied by shrinkage and death of hematopoietic organs [194]. At subcellular level, *in ovo* blood sampling enables serum analysis, including enzyme activity, glucose, blood urea nitrogen levels, and total protein concentration. Interestingly, no major effects were reported after exposure to carbon NPs [202, 203, 224].

The CAM model is also a key system for evaluating oxidative stress induced by NPs, owing to its conserved enzymatic and non‐enzymatic antioxidant defenses [228]. Comparable studies have been performed in *D. melanogaster*, *H. vulgaris, C. elegans*, and zebrafish, which share substantial genomic similarities with humans, particularly in oxidative stress pathways [229, 230]. Oxidative stress, defined as an imbalance between ROS production and neutralization, leads to cellular damage. It can be assessed by measuring ROS production directly (e.g., dichloro‐dihydro‐fluorescein diacetate ‐DCFH‐DA‐ assay, nitroblue tetrazolium assay, etc.) [184, 192, 222], lipid peroxidation (thiobarbituric acid‐based assays, synchrotron Fourier‐transform infrared microscopy) [203, 222, 231], ROS scavenger levels (e.g., non‐protein thiols) [185, 222], CAT activity [222], and the expression of oxidative stress‐related genes [161, 177, 184, 231]. Gene expression analyses have also been applied in the CAM model for vascular studies [202], in *H. vulgaris* for genotoxicity studies (e.g., caspase, antistasin, stress‐responsive genes, p53) [127, 185, 186], and in *D. melanogaster* and *C. elegans*, often in combination with the Comet assay [177, 184].

Taken together, these bridging models highlight complementary strengths and limitations in nanotoxicity research. While CAM remains the reference for vascular and tissue‐level studies, bridging models such as *Hydra spp*., *C. elegans*, *D. melanogaster*, zebrafish, and *B. mori* provide valuable insights at the behavioral, developmental, or molecular scale. Each system offers unique advantages—ranging from accessibility and cost‐effectiveness to genetic tractability—but also inherent constraints that restrict their use to specific endpoints. Rather than serving as stand‐alone solutions, these models form a complementary toolbox that enables a multi‐scale assessment of nanotherapeutics, from organismal survival to subcellular mechanisms.

### 4.2 Evaluating the Intrinsic Therapeutic Effects of Nanoparticles

In addition to the evaluation of the toxicity and the biocompatibility of NPs, bridging models can also be used to assess their therapeutic effect for various diseases, including cancer in *in ovo* models, but also neurodegenerative disorders in *D. melanogaster*, or mitochondrial diseases in *C. elegans*. Indeed, plain NPs can exert intrinsic biological activities that may contribute to or interfere with therapeutic outcomes (Table 3).

**TABLE 3:** Intrinsic therapeutic activities of nanoparticles assessed in bridging models.

Biological activity	Model	Type of NPs	References
Anticancer / pro‐apoptotic	CAM	Ag, Au, Fe_3_O_4_, CuO	[232, 233, 234, 235, 236, 237]
Antiangiogenic	CAM	Carbon allotropes, ZnO, Au	[233, 239, 240, 241, 242, 243, 244, 245, 246, 247]
Pro‐angiogenic	CAM	Cu, Au	[248, 249, 250, 251, 254]
Neuroprotective / mitochondrial diseases	*D. melanogaster*	CeO_2_	[255]
	*C. elegans*	Pt	[256]

The CAM model has become a practical *in ovo* platform to test the intrinsic antitumor activity of NPs in tumor xenografts. Across studies, several metallic and carbon NPs were found to suppress tumor growth via apoptosis and anti‐proliferative effects, e.g., Ag NPs in glioblastoma (GBM) [232], and aqueous‐synthesized carbon QDs [233], Pt NPs [234], and folate‐decorated CuO nanowires in breast cancer [235]. The CAM model has also been used to demonstrate that modifying NP composition and surface chemistry can enhance antitumor effects; Fe_3_O_4_ NPs, which induce apoptosis in mesothelioma cells, exhibited additional anti‐proliferative effects when fabricated with a salicylic‐acid shell [236]. Surface functionalization further modulates therapeutic outcome: PS NPs functionalized with amino groups (NH_2_), but not with carboxyl groups (COOH), inhibited proliferation of leukemia cells grafted on the CAM, and exhibited anti‐angiogenic effects [237]. Consistently, another study showed that intravenously administered PS‐NH_2_ targeted prostate tumor xenografts, whereas PS‐COOH accumulated in the embryonic liver [238].

The CAM model is a gold standard for evaluating antiangiogenic activity of NPs and investigating underlying mechanisms. Using this model, multiple studies have revealed that NPs can inhibit neovascularization *in ovo* through several complementary mechanisms. A common pathway, observed with carbon NPs [239, 240, 241], Au NPs [242, 243], ZnO NPs [244, 245], and cationic poly‐L‐lysine dendrimers [246], involves interference with the fibroblast growth factor 2 (FGF2) / vascular endothelial growth factor (VEGF) axis, thereby reducing endothelial motility. In particular, peptide‐modified Au NPs have been shown to block VEGFR1 signaling and VEGF165‐induced migration [243]. Another inhibitory mechanism, based on endothelial cytotoxicity and impaired tubulogenesis, was also evidenced in the CAM model with Ag NPs in a dose‐dependent manner [247], and with cationic poly‐L‐lysine dendrimers [246].

Beyond identifying antiangiogenic effects, the CAM model also enables the exploration of opposite outcomes, revealing how NP structure can shift the biological response toward angiogenesis rather than inhibition [248, 249, 250, 251]. For example, a comparative study of pristine carbons showed distinct behaviors: fullerene was pro‐angiogenic, whereas diamond NPs and multi‐wall nanotubes exhibited anti‐angiogenic properties, and graphite and graphene NPs showed no effect [252]. Effective modulation of angiogenesis has also been demonstrated by coating Au NPs with peptides known to either stimulate or inhibit arteriogenesis [253]. While undesirable in oncology, pro‐angiogenesis can be leveraged for regenerative purposes; for example, polycaprolactone (PCL) scaffolds incorporating Y_2_O_3_ NPs promoted cell adhesion and vascularization in CAM, supporting tissue‐engineering applications [254]. Together, these findings highlight the CAM model as both a stringent screen for undesired pro‐angiogenic effects of NP formulations in oncology and a valuable platform for harnessing pro‐vascularization for tissue‐engineering applications.

Beyond oncology, other bridging models have also revealed the potential of NPs to modulate oxidative and mitochondrial pathways relevant to neurodegenerative and metabolic disorders. In *D. melanogaster*, CeO_2_ NPs extended lifespan and improved locomotor performance by scavenging free radicals and reducing oxidative stress, suggesting potential applications in the treatment of age‐related neurodegenerative diseases [255]. In *C. elegans*, platinum NPs functionalized with a trans‐acting activator of transcription (TAT) fusion peptide were internalized into mitochondria and restored nicotinamide adenine dinucleotide (NADH) oxidase activity in a complex I–deficient strain, effectively rescuing mitochondrial dysfunction [256]. These findings demonstrate how nanoscale redox‐active materials can mimic or replace defective enzymatic functions in vivo, paving the way for nanotherapies targeting mitochondrial and neurodegenerative pathologies.

### 4.3 Bridging Models to Test Nanoparticles as Drug Delivery Systems

**FIGURE 12:**
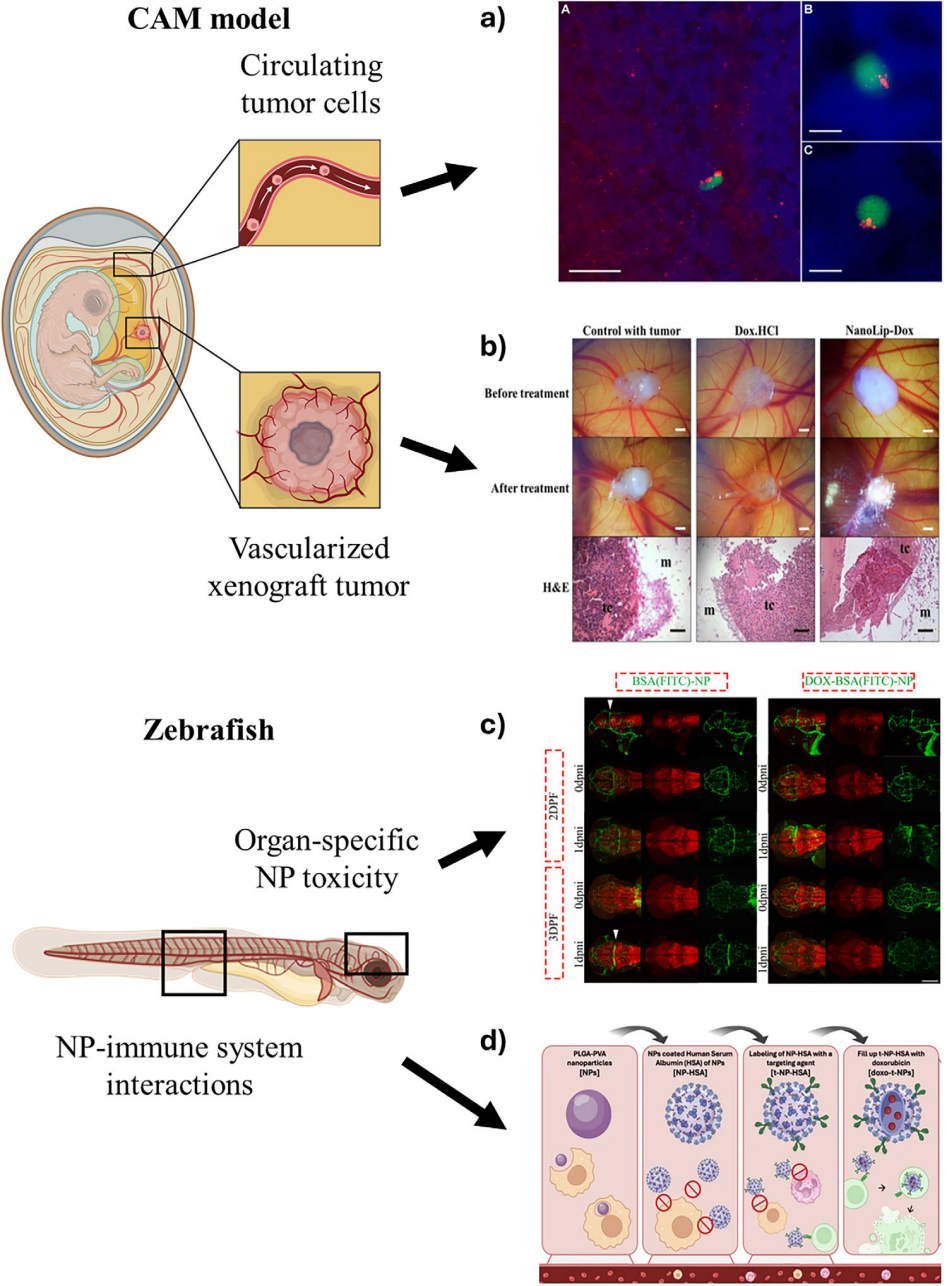
Representative examples of the use of the CAM model and the zebrafish for evaluating NPs as drug delivery systems. (a) Testing of engineered NPs (red) targeting circulating leukemia cells (green) in the embryonic vasculature [262], (b) comparative study of the antitumor effects of nanoencapsulated DOX vs. free drug in breast tumors xenografts on the CAM [265], (c) assessment of brain toxicity of DOX‐loaded engineered NPs injected into zebrafish vasculature [284], (d) evaluation in zebrafish of a therapeutic strategy based on modifying the protein corona composition of DOX‐loaded NPs to prevent macrophage‐mediated elimination after vascular injection [282]. Adapted with permission from [262, 265, 282, 284].

NPs for nanotherapies are designed to increase drug exposure at disease sites by improving solubility/stability, prolonging circulation time, enhancing targeting, and enabling triggered release and theranostics. The CAM and zebrafish models provide rapid and complementary readouts for assessing NP targeting, retention, tissue penetration, efficacy with respect to free drugs, vascular effects, and acute safety: some representative examples are presented in Figure 12.

The CAM model has been widely used for the evaluation of anticancer nanotherapies, owing to the possibility of injecting NPs directly into the vasculature or in the tumor to assess internalization and antitumor effects in xenografts. In breast cancer, several strategies have been tested to specifically target malignant over non‐malignant cells by functionalizing NPs with ligands for overexpressed molecular markers such as intercellular adhesion molecule‐I (ICAM‐1) [257], folate receptors [258], or somatostatin receptor 2 (STTR2) [259]. Functionalization with glucose moieties has also enabled the selective targeting of breast cancer stem cell populations, exploiting their glycolytic phenotype [260]. Similar approaches have been evaluated in invasive thyroid cancer cells expressing folate receptors, where MSNs functionalized with the folate antagonist methotrexate and loaded with fingolimod significantly reduced the invasive behavior of thyroid‐derived xenografts and induced higher levels of apoptosis and necrosis compared to tumors treated with the free drug cocktail [261].

The CAM model has further been applied to target circulating leukemia cells expressing the epidermal growth factor receptor (EGFR), by injecting cells into the embryo vasculature, followed by administration of mesoporous silica NPs (MSNs) encapsulated within a supported lipid bilayer and functionalized with anti‐EGFR monoclonal antibodies [262]. These NPs selectively bound leukemic cells while avoiding endothelial cell uptake. NPs described in these studies were loaded with chemotherapeutic agents such as quercetin, diacerein, fingolimod and gemcitabine, which induced cytotoxic effects and enhanced apoptosis. The CAM model was also used to demonstrate that nanoformulations can outperform their free‐drug counterparts, particularly in the case of doxorubicin (DOX), evaluated on xenografts of breast cancer [263, 264, 265], melanoma, and fibrosarcoma [266]. Higher local concentrations and improved intratumoral diffusion of the drugs were observed in these xenografts [263, 264, 265, 266], along with lower embryonic toxicity [265], resulting in a markedly enhanced antitumor efficacy compared to free DOX at similar concentrations. Other drugs, such as paclitaxel, lapatinib, and gemcitabine, also showed higher antitumor efficiency when loaded into engineered NPs, in ovarian [267], breast [268], and prostate cancer models [269], respectively. However, the encapsulation of certain agents did not necessarily translate into superior therapeutic efficacy: for example, cannabidiol (CBD)‐loaded poly‐lactic‐co‐glycolic acid (PLGA) NPs exhibited only limited improvement with respect to the free drug, underscoring the relevance of bridging models for the preliminary evaluation and selection of promising nanoformulations [270].

CAM readouts are highly sensitive to vascular alterations, enabling the assessment of anti‐angiogenic drug effects when delivered through NP‐based systems. The impact of anti‐angiogenic drugs encapsulated in engineered NPs targeting integrin receptors, which are overexpressed in a lot of tumor cells and vascular endothelial cells, has been evaluated on the embryo vasculature [271, 272, 273, 274]. For instance, MSNs decorated with Arg‐Gly‐Asp acid (RGD) peptides, recognized by integrin receptors, and loaded with imidozolium trans‐imidazoledimethyl‐sulfoxidetetrachlororutheate (NAMI‐A), effectively suppressed VEGF‐induced angiogenesis in CAM model [271]. Several other drugs, including 5‐fluorouracile, oridonin, ursolic acid, vincristine, and DOX, known for their anti‐angiogenic activity, confirmed their ability to reduce neovascularization when formulated into nanostructures, thereby enhancing their antitumor effects [275, 276, 277, 278, 279]. Interestingly, comparable outcomes were achieved with Ag NPs synthesized using a methanolic extract of *Praecitrullus fistulosis*, a vegetable fruit, opening new perspectives for green‐synthesized NPs [280]. Finally, a retinoblastoma xenograft model was used to evaluate the anti‐angiogenic potential of atrial natriuretic peptide (ANP)‐conjugated hyaluronic acid‐coated Au NPs directly on tumor vasculature, demonstrating a marked reduction in both angiogenesis and tumor growth [281].

Building on the insights gained from the CAM model, the zebrafish model offers a complementary vertebrate system with closer physiological relevance to mammals, characterized by conserved angiogenic and immune pathways. This model was used to validate a strategic therapeutic approach based on the covalent attachment of human serum albumin (HSA) to polymeric NPs loaded with DOX, aiming to modify the protein corona composition and minimize immune recognition. The HSA coating prevented opsonization and subsequent macrophage‐mediated clearance, while surface decoration with anti‐CD19 antibodies enabled specific targeting of acute lymphoblastic leukemia xenografts in zebrafish, resulting in efficient DOX delivery, tumor growth suppression, and increased survival [282].

The zebrafish model can also be exploited to investigate systemic and organ‐specific responses to anticancer nanotherapies. For instance, ponatinib, a tyrosine kinase inhibitor used in chronic myeloid leukemia, is associated with severe cardiotoxicity, which limits its clinical utility. When encapsulated in polymeric NPs and tested in zebrafish cancer xenograft models, ponatinib exhibited markedly reduced systemic and cardiac toxicity, although tumor size reduction was limited, likely due to lower cellular internalization compared to the free‐drug [283]. Zebrafish have also been employed to establish circulating tumor cell (CTC) xenograft models, where BSA NPs loaded with DOX effectively localized to and eliminated breast CTCs [284]. Interestingly, folic acid decoration accelerated NP‐tumor colocalization but did not improve therapeutic efficacy, highlighting that targeting kinetics does not necessarily correlate with therapeutic benefit [284].

Finally, zebrafish breast cancer xenograft models have been used to explore tunable drug‐release kinetics, for example using cisplatin‐loaded hydroxyapatite NPs, stabilized by citrate and exhibiting pH‐dependent dissolution, which effectively reduced cancer cell viability in a manner comparable to free cisplatin [285]. Similar comparisons were made with DOX‐loaded formulations: DOX‐loaded peptide‐modified liposomes achieved enhanced targeting and tumor suppression in HeLa xenografts [286], while DOX‐loaded cross‐linked Pluronic polymeric micelles showed greater accumulation in metastatic melanoma cells and superior anticancer efficacy relative to free DOX [287].

In summary, both the CAM and zebrafish models serve as robust and complementary bridging platforms for the preclinical assessment of NP‐based drug delivery systems, providing valuable insights into biodistribution, vascular targeting, efficacy, and safety prior to evaluation in mammalian models. Building on these foundations, the next Section explores innovative NP‐based therapeutic strategies, including photodynamic, photothermal, and magnetothermal approaches. These modalities exemplify how nanocarriers can be engineered not only to deliver drugs efficiently but also to integrate physical and biochemical functions, enabling multimodal and precision therapies that can be effectively evaluated using alternative models.

### 4.4 Innovative Nanoparticle‐Based Therapeutic Strategies

**FIGURE 13:**
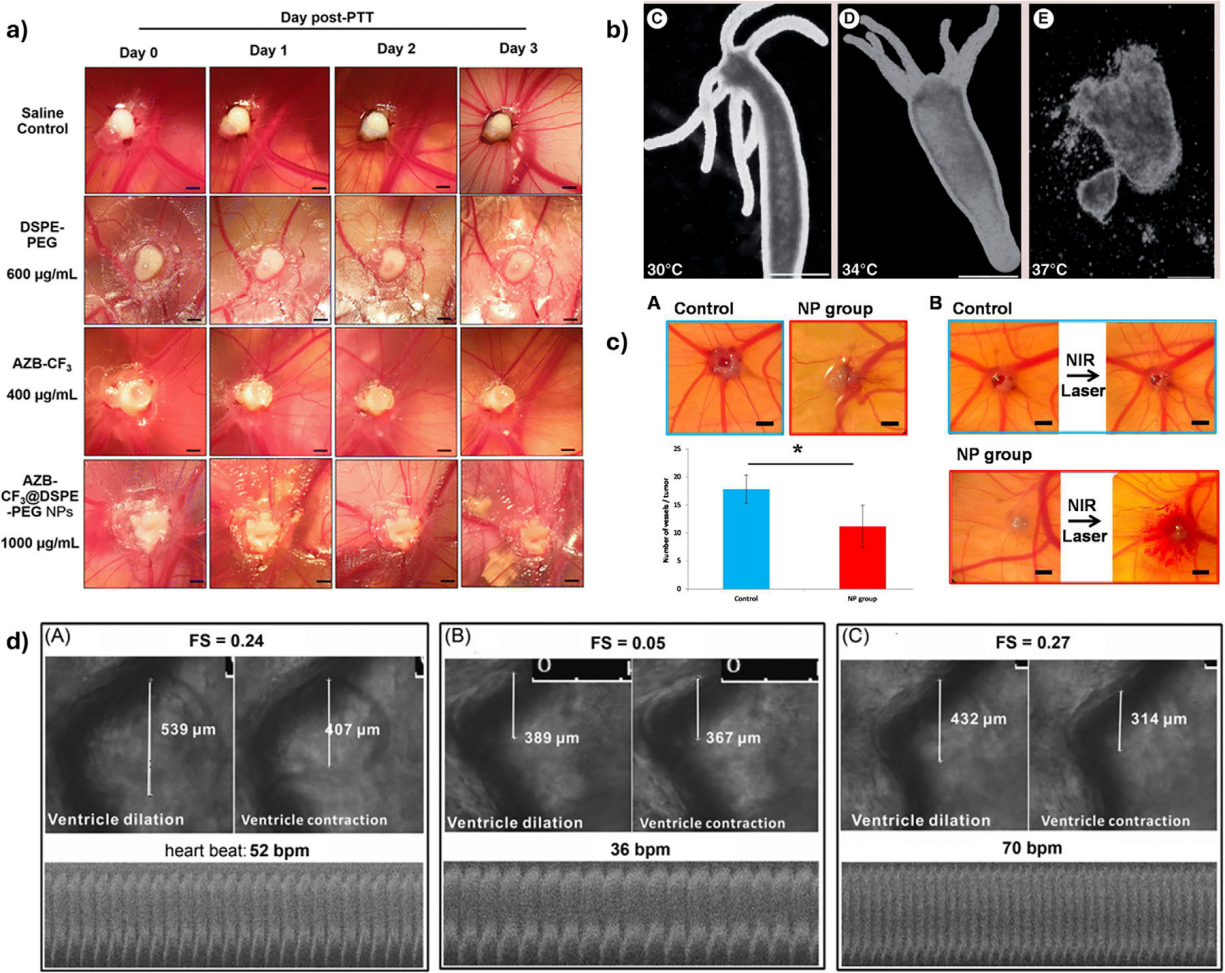
Representative examples of studies using bridging models for the development of innovative nanotherapies. (a) Aza‐BODIPY‐based polymeric NPs for photothermal cancer therapy evaluated on breast tumor xenograft in the CAM [303], (b) effects of heat shock on *H. vulgaris* morphology in studies investigating NP‐based magnetic hyperthermia therapies [124], (c) effects of NP‐based multimodal therapies combining PTT, PDT and two anti‐vascular drugs on tumor xenografts in the CAM [310], (d) therapeutic results of photothermally controlled curcumin delivery in zebrafish larvae for heart failure treatment [307]. Adapted with permission from [124, 303, 307, 310].

Following the demonstration of NP‐mediated drug delivery and efficacy in bridging models, recent research has focused on innovative therapeutic strategies that exploit the unique physicochemical properties of NPs to achieve controlled, localized, and multimodal treatment effects. These emerging approaches include photodynamic therapy (PDT), photothermal therapy (PTT), and magnetothermal therapy, which leverage light or magnetic energy to selectively damage tumor tissues or trigger on‐demand drug release. Bridging models—primarily CAM but also *H. vulgaris*., *D. melanogaster*, zebrafish, and *C. elegans—*provide rapid, information‐rich readouts of these modalities (Figure 13).

PDT relies on a photosensitizer that, upon light exposure in the presence of oxygen, generates ROS to damage tumor cells and/or tumor vasculature [288]. NPs improve PDT by stabilizing hydrophobic photosensitizers, prolonging intravascular residence, tuning drug–light intervals, and biasing uptake toward tumor or endothelium. PTT uses mostly NIR‐absorbing nanoagents to transform light into heat for hyperthermia or ablation, and the same photothermal effect can be harnessed to trigger on‐demand drug release [288]. Magnetothermal approaches extend this concept by stimulating magnetic NPs with alternating fields, enabling local hyperthermia or remote biological actuation [288]. To translate these physical principles into practical nanotherapies, numerous studies have explored how NP‐based carriers can enhance the delivery and performance of photosensitizers in biological systems.

NPs are increasingly used as photosensitizers delivery systems to enhance solubility, bioavailability, and selectivity, and their effects on vasculature and tumor tissue are best captured in bridging models such as the CAM. Owing to its transparent, highly vascularized structure and ease of imaging, this system allows real‐time visualization of photodynamic responses at both tissue and microvascular levels, linking formulation parameters to biological outcomes. In this context, nanocarriers have proven instrumental in optimizing vascular‐targeted PDT. Small or lipophilic NP formulations improved photosensitizer retention and photothrombotic efficiency [289, 290], while PEGylated and PLGA‐based carriers prolonged intravascular residence and enhanced vascular occlusion compared with free drugs [291, 292]. The photosensitizer location within liposomal bilayers also dictated performance: porphycenes embedded deep in the bilayer were inactive in vitro but induced significant endothelial damage in vivo [293]. Stable tetraether‐lipid vesicles further enabled selective anti‐angiogenesis without harming quiescent vessels [294]. By analyzing CAM readouts such as hemorrhage, thrombosis, and vessel lysis, these models provide a precise and high‐throughput evaluation of vascular PDT selectivity and safety [295, 296, 297].

Beyond vascular assessment, the CAM platform facilitates evaluation of antitumor efficacy and microenvironmental modulation. Lipoprotein‐based systems delivering hypericin, liposomal meso‐tetra(hydroxyphenyl)chlorin (m‐THPC, Fospeg), and nebulizable or tetraether‐lipid curcumin formulations demonstrated improved tumor selectivity, sustained release, and reduced off‐target toxicity [296, 298, 299, 300]. In GBM xenografts, curcumin‐ and parietin‐loaded lipid NPs decreased tumor growth and angiogenesis assessed through positron emission tomography/computed tomography (PET/CT) and histological validation [301]. Altogether, bridging models such as the CAM uniquely integrate vascular and tumor‐level endpoints, enabling comprehensive evaluation of NP‐mediated photodynamic effects that would be difficult to capture in conventional systems.

Bridging models have also been instrumental in advancing PTT and heat‐triggered nanomedicine. In avian systems, the CAM enables real‐time evaluation of both tumor ablation and vascular damage under NIR irradiation. Au NPs functionalized with anti‐angiogenic peptides produced localized cauterization and potent inhibition of VEGFR signaling [302], while aza‐boron‐dipyrromethenes (BODIPY) polymeric NPs combined strong NIR absorption with efficient tumor suppression and anti‐metastatic effects in breast cancer xenografts [303]. Polydopamine NPs loaded with sorafenib and coated with cancer‐cell membranes extended these applications to liver cancer, demonstrating selective targeting and combined chemo‐photothermal efficacy in *ex ovo* quail embryos [304].

In parallel, invertebrate and aquatic models have provided key mechanistic insights into photothermal action and controlled drug release. In *H. vulgaris*, Au nanoprisms induced precise, NIR‐triggered cell ablation accompanied by heat‐shock gene activation, supporting their use as biocompatible thermal mediators [305]. Recent work further demonstrated that heat‐triggered nanomedicine can be extended beyond tumor ablation to trigger cellular activity modulation. Using polydopamine NPs irradiated by NIR light, a localized photothermal activation of neuron‐like cells was achieved, inducing calcium transients and neurotransmitter release in ex vivo *D. melanogaster* brains. This study underscores the potential of biocompatible photothermal nanomaterials to remotely and reversibly regulate cellular activity with high spatial and temporal precision [306].

In zebrafish, MSNs functionalized with molecular rotaxanes achieved light‐ and heat‐dependent release of curcumin, effectively reversing cardiac dysfunction in vivo [307]. Collectively, these models capture complementary levels of biological organization, from molecular stress pathways to tissue‐level thermotherapy, demonstrating how alternative organisms enable integrated, ethically feasible evaluation of NM heating, targeting, and therapeutic control that would be difficult to achieve in conventional mammalian systems.

Alternative in vivo models play a pivotal role in elucidating the biological mechanisms underlying magnetothermal stimulation. In *H. vulgaris*, exposure to magnetic NPs under alternating magnetic fields triggered heat shock protein 70 (Hsp70) transcription without measurable macroscopic heating, revealing conserved thermotolerance pathways and validating Hsp70 as a molecular marker of sublethal hyperthermia [124]. In *C. elegans*, phosphatidylcholine‐coated magnetic NPs combined with a static magnetic field altered lipid metabolism and β‐oxidation gene expression, indicating systemic metabolic adaptations to magnetic stimuli [308]. By revealing how living systems respond to nanoscale heat and magnetic fields, these bridging models provide mechanistic insight that supports the rational design of future therapeutic platforms. Building on this mechanistic foundation, recent efforts have shifted toward multimodal nanoplatforms that integrate complementary physical and pharmacological mechanisms—combining photothermal, photodynamic, and chemotherapeutic actions within a single carrier to achieve synergistic efficacy and vascular selectivity.

The development of multifunctional nanocarriers capable of integrating several therapeutic modalities has opened new avenues for precision therapy testing in bridging models. Hexagonal Au nanorings demonstrated strong plasmonic absorption, enabling efficient photothermal ablation of melanoma cells in the CAM while simultaneously generating ROS and disrupting mitochondrial potential, thereby merging oxidative and thermal cytotoxicity in the absence of external photosensitizers [309]. Similarly, an RGD‐targeted nanosystem co‐delivering an antiangiogenic and a vascular‐disrupting drug, incorporated photothermal and photodynamic functions by embedding Au nanorods and a photosensitizer (indocyanine green) [310]. When tested on *ex ovo* CAM fibrosarcoma xenografts, this composite platform induced extensive vascular destruction and vessel collapse upon NIR irradiation, demonstrating how combined anti‐vascular strategies can achieve high efficacy with spatial precision. These examples highlight how bridging models such as the CAM uniquely enable dynamic, high‐resolution assessment of multimodal nanotherapeutics, capturing tumor–vasculature interactions, local heating, oxidative stress, and drug release within the same living system. As a result, they form an indispensable link between mechanistic understanding and the preclinical optimization of complex therapeutic nanoplatforms.

### 4.5 Toward Next‐Generation Nanomedicine

Collectively, presented studies demonstrate how bridging models—ranging from *H. vulgaris* and *C. elegans* to zebrafish and the avian CAM—serve as indispensable platforms to unravel how NMs interact with biological systems at molecular, cellular, and organism levels. Their transparency, rapid development, and ethical accessibility enable real‐time analysis of vascular dynamics, oxidative stress, metabolic adaptation, and NP distribution under controlled light or magnetic stimuli. By integrating multiple readouts within the same organism, these systems bridge mechanistic understanding with translational assessment, accelerating the development of safer and more effective nanotherapies while reducing reliance on higher‐order animal models.

This approach extends naturally to next‐generation multifunctional and radiosensitizing nanoplatforms. The ultrasmall‐in‐nano architectures encapsulating a cisplatin prodrug and tested in CAM pancreatic models exemplify this continuum: by coupling controlled drug release with ionizing radiation, they enhanced DNA damage and apoptosis, achieving superior antitumor effects [311]. Overall, bridging models represent a crucial experimental interface among NM design, therapeutic innovation, and sustainable preclinical validation.

## 5 Discussion

The review of nanotechnology's health and environmental impacts highlights an unavoidable paradox: the nanoscale features that make NMs transformative are the same characteristics that can drive toxicity and ecological disruption. Their enhanced surface area, chemical reactivity, and ability to cross biological barriers enable unprecedented therapeutic and industrial applications, yet also create avenues for unintended interactions at molecular, cellular, and organism level. Metallic NPs such as Ag and TiO_2_ can generate ROS, leading to oxidative stress and DNA damage [31, 312], while carbon nanotubes may induce fibrotic responses reminiscent of asbestos exposure [313]. Similarly, NPs that are highly effective as antimicrobial coatings in industry, like Ag or ZnO, may persist in aquatic ecosystems, dissolve to release toxic ions, and disrupt microbial communities or phytoplankton [55, 314, 315]. These examples underscore the necessity of establishing a rigorous and precautionary framework that reconciles technological innovation with environmental and human health protection, thereby ensuring that the long‐term trajectory of nanotechnology remains both ethically responsible and ecologically sustainable.

A recurring insight from the literature is that no single experimental system can provide comprehensive information about the risks and benefits of NMs. In vitro assays allow detailed study of NP–cell interactions, such as oxidative stress induction or genotoxicity, but they fail to capture complex organismal responses like biodistribution, immune activation, or reproductive effects. Mammalian in vivo models, especially rodents, remain the gold standard for translational assessment, and they have been critical in bringing nanocarriers such as lipid NPs for mRNA vaccines to the clinic [316]. However, their scalability is limited by ethical concerns, financial cost, and time constraints. Bridging models—including invertebrates like *Hydra spp*., planarians, *C. elegans*, and *D. melanogaster*, as well as vertebrates such as zebrafish and avian embryos—occupy a middle ground, integrating multicellular and systemic responses in ways that are experimentally tractable and ethically defensible. For instance, *H. vulgaris* has been employed to study how CdTe QDs induce progressive morphological changes before death, linking NP uptake to whole‐body regenerative failure [185], while zebrafish embryos have provided real‐time visualization of neurobehavioral effects such as hyperactivity after chitosan NP exposure [189]. These cases illustrate how bridging models complement traditional assays by uncovering organism‐level outcomes that cannot be captured in simple cell cultures.

Such systems also provide essential insights into ecotoxicology. Marine bivalves like *Mytilus galloprovincialis*, act as sentinel species, filtering large volumes of water and accumulating NPs, thereby revealing how NPs affect immune and reproductive processes [317]. Planarians and *Hydra spp*., with their extraordinary regenerative capacities, highlight how NM exposure interferes with stem‐cell‐driven morphogenesis, providing sensitive indicators of developmental toxicity [318]. Zebrafish embryos, with their optical transparency and rapid development, enable researchers to trace the consequences of NP exposure on organogenesis, vascular formation, and behavior [319]. Collectively, these models demonstrate that environmental effects cannot be disentangled from human health risks: NPs released through industrial discharge, consumer products, or agriculture inevitably enter ecosystems where they bioaccumulate and biomagnify through trophic chains, eventually returning to humans via food and water: [320, 321, 322] the ecological and biomedical aspects of nanotechnology are thus inherently interlinked.

Beyond risk assessment, bridging models also play a critical role in advancing nanomedicine. The CAM assay, a widely used *in ovo* system, has become a powerful platform for studying angiogenesis, vascular toxicity, and tumor–vasculature interactions. For example, the HET‐CAM test has been extensively applied to lipid and polymer NPs intended for ocular or dermal delivery, providing rapid readouts of vascular irritation, coagulation, and hemorrhage [323]. The CAM has also enabled testing of anticancer nanotherapies in various xenograft models of human cancer, like triple negative breast cancer (TNBC), leukemia and GBM [232, 235, 237]. Similarly, zebrafish embryos have been exploited to evaluate biodistribution and targeting, as in studies where albumin‐coated NPs delivered DOX specifically to leukemia cells, or where platinum‐loaded hydroxyapatite NPs reduced breast cancer cell survival through controlled release [282, 285]. Invertebrate systems extend these applications: *D. melanogaster* has been used to study how CeO_2_ NPs protect against oxidative stress in models of neurodegenerative disease, while *C. elegans* carrying mitochondrial mutations showed functional recovery after exposure to platinum NPs functionalized for mitochondrial uptake [256, 324]. Together, these examples highlight how bridging organisms not only reveal toxic outcomes, but also help identify the intrinsic therapeutic potential of certain NPs, whether anticancer, antiangiogenic, or neuroprotective.

Despite these advantages, the interpretation of findings across models remains complex. NP properties such as size, charge, dissolution rate, and surface functionalization critically shape biological responses, yet inconsistent characterization and reporting limit reproducibility [325, 326]. The same material may behave differently depending on whether it is administered through diet in *D. melanogaster*, immersion in zebrafish water, or surface deposition on the CAM. Such variability complicates extrapolation to human exposure scenarios. Moreover, while pathways like oxidative stress and apoptosis are conserved across species, mammalian‐specific features of immunity or endocrine regulation may not be fully captured. For instance, while *Hydra spp*. and zebrafish embryos provide powerful insights into developmental processes, they cannot fully recapitulate adaptive immune responses relevant for nanomedicine safety [327, 328]. Without harmonization of experimental metrics, including standardized dose reporting (per mass, particle number, or surface area) and consistent particle characterization, bridging models risk producing fragmented datasets that slow regulatory uptake and practical application.

Nonetheless, the collective evidence strongly supports embedding these models within an integrated, multi‐tiered framework. By combining high‐throughput in vitro assays, diverse invertebrate and vertebrate bridging systems, and selective mammalian studies, researchers can build layered evidence that captures NP effects from molecular to organism scales. This approach not only reduces reliance on higher‐order animals, in line with global ethical imperatives, but also accelerates the development pipeline for nanotherapeutics and strengthens ecological risk assessment. Importantly, it reflects the “One Health” perspective, recognizing the deep interconnection between human health, animal welfare, and ecosystem integrity. The broader implications of this shift extend beyond laboratory practice. The adoption of bridging models represents a regulatory and cultural transition toward sustainable innovation. EU directives and OECD guidelines increasingly encourage the reduction of vertebrate testing, and bridging models directly respond to this call by offering robust, ethically feasible alternatives [329, 330, 331, 332, 333]. In translational medicine, they enable safer‐by‐design approaches, where potential hazards are identified and mitigated early in NP development rather than after environmental release or clinical translation. By revealing both promise and peril in parallel, bridging models contribute not only to scientific progress but also to public trust in nanotechnology, which is critical for its long‐term success.

In summary, bridging models are indispensable for addressing the dual impact of nanotechnology. They cannot replace traditional systems entirely, but their ability to capture intermediate levels of complexity while remaining accessible, scalable, and ethically acceptable makes them powerful allies in both risk assessment and therapeutic innovation. As illustrated by the diverse examples of *Hydra spp*., planarians, bivalves, zebrafish, *D. melanogaster*, *C. elegans*, and CAM assays, each model provides unique insights into how NPs interact with living systems. By placing them within an integrated framework, researchers can better anticipate risks, enhance translational success, and guide nanotechnology toward a safer and more sustainable trajectory.

## 6 Conclusions and Perspectives

Nanotechnology continues to advance at a remarkable pace, but its dual impact on health and environment highlights the need for experimental approaches that are both predictive and sustainable. Alternative bridging models have already shown their value in revealing organism‐level outcomes across biomedical and ecological domains, yet their greatest potential lies ahead as part of a more integrative and forward‐looking framework.

A key priority for future progress is the establishment of standardized and harmonized methodologies: the lack of consistency in NP characterization, dose metrics, and exposure conditions remains a major barrier to comparability across studies. Developing shared protocols for reporting NP properties and biological endpoints will improve reproducibility and accelerate regulatory acceptance of bridging models. Building upon this standardized framework, expanding the molecular and analytical capabilities of these systems will be essential. Advances in omics technologies, genome editing, and high‐resolution imaging can transform models such as zebrafish, *C. elegans*, and CAM into even more powerful platforms for dissecting the mechanisms of NM interactions. These tools will also strengthen the predictive link between alternative models and human health outcomes.

Bridging models are equally poised to play a central role in next‐generation nanotherapeutics. Beyond testing safety, they can guide the design of multifunctional nanoplatforms for photodynamic, photothermal, and magnetothermal therapies, as well as targeted drug delivery systems. Integrating these models into early‐stage pipelines will enable rapid screening of therapeutic efficacy alongside toxicity, supporting safer‐by‐design approaches that minimize failure in late‐stage trials. The integration of environmental dimension into this framework must moreover remain central. Bridging species such as *Hydra spp*., planarians, and bivalves should be incorporated into ecological monitoring frameworks to assess NP persistence and bioaccumulation in real‐world contexts. Linking these findings with biomedical models reflects the “One Health” perspective, ensuring that human well‐being is considered alongside ecosystem integrity.

Another key point is that broader adoption of these approaches can yield substantial financial benefits by reducing reliance on resource‐intensive vertebrate studies. For example, published cost/animal‐use estimates for repeated‐dose vertebrate assays indicate that a 28‐day rodent study (OECD TG 407) typically involves ∼40 animals and ∼49 k€ in direct study costs, while longer studies (e.g., 90‐day) require more animals and higher costs. When alternative models are used upstream for screening and prioritization, fewer candidate materials progress to these higher‐cost in vivo phases, improving overall R&D efficiency.

Also, from a 3Rs standpoint, these models contribute to Replacement by providing non‐vertebrate or non‐animal platforms for early hazard identification and mechanistic hypothesis testing; to Reduction by enabling tiered strategies that triage low‐concern materials before vertebrate testing; and to Refinement by informing dose selection and endpoint prioritization when vertebrate studies remain necessary, thereby avoiding poorly targeted or repeated experiments. At a program level, prior analyses of regulatory testing demand have emphasized that vertebrate‐based requirements can translate into very large animal numbers and costs, reinforcing the value of scalable high‐throughput approaches. Finally, the field would benefit from sustained interdisciplinary collaboration. Material scientists, toxicologists, clinicians, and regulators must work together to embed bridging models into global testing strategies: such collaboration will be essential to move beyond proof‐of‐concept studies toward validated, widely accepted methodologies that support innovation while addressing ethical and ecological concerns.

In summary, the future of nanotechnology depends not only on innovation, but also on the capacity to evaluate safety and efficacy with approaches that are scientifically robust, economically efficient, and aligned with societal expectations for animal welfare and environmental stewardship. Bridging models—ranging from invertebrates to small vertebrates—represents a cornerstone of this effort. By embracing standardization, expanding molecular capabilities, and integrating ecological and biomedical insights, these models can shape a new era of nanotechnology that is safer, more sustainable, and firmly aligned with societal and environmental needs.

## Author Contributions

M.C.L. contributed to conceptualization, visualization, writing of the original draft, and writing, review, and editing. M.Ber. contributed to conceptualization, visualization, and writing of the original draft. M.Bat. contributed to the visualization and writing of the original draft. G.C. contributed to conceptualization, supervision, project administration, resources, and writing – review and editing.

## Conflicts of Interest

The authors declare no conflicts of interest.

## Data Availability

The authors have nothing to report.
